# Toward More-Than-Human Understandings of Sport and the Environment: A New Materialist Analysis of Everyday Fitness Practices

**DOI:** 10.3389/fspor.2021.660935

**Published:** 2021-06-01

**Authors:** Julie E. Brice, Holly Thorpe

**Affiliations:** Te Huataki Waiora School of Health, Hamilton, New Zealand

**Keywords:** entanglement, feminist new materialisms, environment, activewear, Karen Barad, agential realism, fitness

## Abstract

Sport and fitness have long been linked with healthy lifestyles, yet most sporting events and consumption practices are highly detrimental to the environment. While academics have examined the harmful effects of sporting mega-events and the production and consumption of sport equipment and clothing, there has been less engagement with the “mundane,” everyday activities of consuming, laundering, and recycling of fitness objects. In this paper, we explore the potential in feminist new materialisms for rethinking the complex relationships between sport, fitness, and the environment. In particular, we explain how our engagement with Karen Barad's theory of agential realism led us to rethink women's habitual fitness practices as connected to environmental degradation. Working with Barad's concept of entanglement, we came to notice new human-clothing-environment relationships, focusing on how athleisure clothing itself is an active, vital force that intra-acts with other non-human (and human) matter within the environment. Adopting a diffractive methodology that included reading interviews with women about their activewear practices, our own experiences, new materialist theory, and environmental literature through each other, we focus on two examples that emerged through this process: laundering and disposal practices. Through these examples, we demonstrate the ways in which new materialisms encouraged us to move toward non-anthropocentric understandings of the sport-environment relationship and toward new ethical practices in our everyday fitness lifestyles.

## Introduction

The world is facing a massive environmental crisis with rapid rates of deforestation, increased production of greenhouse gases, rising global temperatures and sea levels, and extensive loss of biodiversity as the Sixth Great Extinction looms (Carrington, 2017). While there are a variety of factors that have led to this alarming state, there is little doubt that human actions and anthropocentric thinking—where human experiences and needs are valued above the non-human—have been major contributors (World Wildlife Fund, n.d.). Recognizing human roles and responsibilities in these processes, many scholars have begun turning toward different ontological and epistemological approaches that view the world through a more connected and relational lens. One such framework is new materialisms, an umbrella term for a range of theories and concepts that are premised on a relational ontology where human and non-human matter are seen as constantly intra-acting and where matter is recognized as lively and agentic. Herein we explore the potential in new materialisms for rethinking the complex relationships between sport, fitness, and the environment.

Many scholars within sport sociology and other leisure and fitness fields have explored the connection between the environment and sport. In particular, there has been a large body of literature interested in the environmental impact of mega-sporting events, as well as scholarship analyzing and critiquing many sporting organizations' sustainability efforts and practices. While this literature is important and necessary, it is often underpinned by assumptions that the environment and non-human matter are passive, acted upon by humans, rather than viewing the environment and non-human matter as active and *entangled* with humans. This literature also tends to focus on larger, macro-scale impacts (mega events, organizations, teams), often ignoring the micro-impacts of sporting and leisure practices. In this paper, we share findings from a larger project using new materialisms, specifically Karen Barad's theory of agential realism, to think about the activewear^1^ phenomenon and women's experiences of fitness. Through agential realism's emphasis on materiality and human-material relationships, during this project, we came to new noticings and understandings of the complex relationship between the everyday wearing of activewear and environmental degradation. Exploring the impact of activewear on the environment was never the main purpose of the research project. Instead these ideas emerged through the agential realist approach to the research process and ways of knowing, thus highlighting the value of more-than-human approaches for prompting us to go beyond anthropocentric understandings of the sport-environment relationship.

Here, we speak to some of these new noticings, primarily drawing upon Barad's concept of entanglement to explore how women's habitual fitness practices and the activewear phenomenon (clothes worn for physical activity) are intimately connected to environmental degradation. In so doing, we reimagine human-clothing-environment relationships, focusing on how athleisure clothing itself is an active force that intra-acts with other non-human (and human) matter within the environment. To accomplish this, we draw upon a range of different qualitative and creative methods (interviews, focus groups, arts-based methods) in addition to our own experiences of consuming, washing and disposing of activewear, alongside an ongoing engagement with environmental research.

This paper begins with a brief section introducing the reader to activewear and research on this growing phenomenon. We then conduct a review of some of the expansive literature around sport and the environment, followed by more specific literature on sporting objects, activewear, and the environment. After reviewing key literature, we introduce new materialist theory, and specifically, Karen Barad's theory of agential realism, as well as review some of the scholars who are drawing upon new materialist theory to explore the connections between sporting culture and the environment. We next provide an overview of our diffractive methodology and analytical approach before exploring two examples to rethink the fitness-sport-environment relationship; (i) laundering of activewear, and (ii) disposal of activewear. Through these examples, we demonstrate the ways in which new materialisms encouraged new noticings in our data and a move toward non-anthropocentric understandings of the sport-environment relationship.

## Feminist Readings of Activewear

Activewear is casual clothing designed for physical activity and often refers to, but is not limited to, stretchy yoga pants/leggings, sweat-wicking synthetic tops, and tight-fitting crop tops. While there is activewear designed specifically for men, it is the women's industry that has grown exponentially over the past 10 years, and therefore, activewear often refers to clothing worn by women (Lipson et al., 2020). While figures vary, one report estimated the global activewear market to be valued at over USD$350 billion (Driver, 2020). Whereas, many other industries have suffered as a result of COVID-19, the athleisure industry continues to grow. In fact, the online sales of many activewear companies, such as ASOS and lululemon, increased between 2019 and 2020, and overall sales of activewear in the UK grew by 17% during this period (Bain, 2020). Part of the reason for activewear's increasing sales over the past decade has been its transition from something worn only in the gym, to clothing acceptable in other social spaces—cafes, grocery stores, schools. Athleisure is now highly visible, including on social media, celebrity culture, advertising, and TV/film.

Recognizing the growing popularity of activewear, many international mainstream apparel retailers—The GAP, American Eagle Outfitters, Primark, Kohls, the Warehouse—have developed their own lines of activewear with other sportswear companies expanding upon their women's lines to include maternity and period-proof activewear. Popular brands such as Adidas, Nike, and Under Armor have all drastically increased the size of their women's apparel lines and spent millions to develop women-centered advertising and marketing campaigns. In 2015, Adidas' head of women's products, Nicole Vollebregt, stated that “Women are the biggest growth opportunity for Adidas,” adding that “our women's business is currently growing faster than men's” (Chitrakorn, 2017).

In response to its prevalence in popular culture, in the past 5 years various scholars have begun to conduct critical analyses of the activewear phenomenon (Lavrence and Lozanski, 2014; Hauff, 2016; Horton et al., 2016; Nash, 2016; Haaksluoto, 2020; Lipson et al., 2020; Brice and Thorpe, 2021a,b). Much of this literature has critiqued the neoliberal, healthism^2^ ideologies within activewear companies' marketing, showing how companies have used concepts of empowerment, independence, self-care, discipline, and bodywork to sell their products (Lavrence and Lozanski, 2014; Horton et al., 2016; Nash, 2016). In a similar vein, but more interested in women's experiences of activewear, some studies have conducted qualitative research to look at the impact of activewear on women's physical activity practices, body confidence, and identity formation (Hauff, 2016; Lipson et al., 2020). A small, but growing body of research is interested in women of color's experiences of activewear and the fitness industry, an industry often critiqued for its whiteness (Azzarito, 2019), as well as how activewear and sportswear are taken up in non-Western countries (Kiuchi, 2021; Saied and Creedon, 2021).

Related research has explored the production of sportswear, examining companies' policies on and practices of labor and human rights. Much of this research was in response to the anti-sweatshop movement of the 1990s—a social movement created to address the exploitation of workers in poorer countries by wealthier countries (Cushman, 1998; Bartley and Child, 2014; Mezzadri, 2016; Williams, 2016). During this time, Nike was a major target of anti-sweatshop campaigns resulting in many scholars conducting analyses of the sportswear company and their human rights policies (Knight and Greenberg, 2002; Greenberg and Knight, 2004; Johnson, 2016). Taking a feminist approach to Nike and their polices, Dworkin and Messner (2002) show how Nike (and other companies) capitalize upon feminist notions of empowerment and independence in their advertising, yet simultaneously are complicit in international systems of gender oppression exploiting a largely poor, female-based workforce to produce their clothing. Importantly, research on activewear often focuses primarily on the wearer of the clothing and/or the social discourses prevalent in the activewear industry, ignoring the large workforce of women in developing countries that are most often responsible for the production of the clothing. Dworkin and Messner (2002) remind scholars of the moving body that “it is not just muscular, or athletic, or ‘fit' bodies that must be considered in women's liberation—it is also laboring bodies” (pp. 22).

In part due to the anti-sweatshop movement popularized in the 1990s, many activewear companies use their websites to publicly discuss their “ethical code of conduct” and efforts made to ensure their production sector is treating employees “fairly.” For example, on Sweaty Betty's website, customers can access their “Modern Slavery Statement” and read about their efforts to ensure “everyone who works for us or who produces goods under the Sweaty Betty brand is treated well and chose said employment freely.” (Sweaty Betty London Modern Slavery Statement, 2019) Similarly, Nike customers can read about their “Human Rights and Labor Compliance Standards” which describe the various steps undertaken by Nike to treat their workers fairly (Nike, 2021). By emphasizing their ethical principles, companies have attempted to prevent the consumer backlash that erupted in the 1990s.

Currently, consumers, and particularly women consumers, have become more concerned with the issues of environmentalism and sustainability. Research has shown that women, more than men, value the environment and are more likely to engage in environmentally friendly behavior (Arora-Jonsson, 2011; Baier et al., 2020). Other research has emphasized the growing popularity of sustainability in consumers' choices when buying activewear. One study looked at customer's willingness to pay for “green goods” by examining Patagonia's (high-end outdoor wear company) switch from conventionally grown cotton to using organically grown cotton. They found that customers are willing to pay premium price for items they perceive to be environmentally friendly even if there is no direct benefit to the consumer (Masanell-Casadesus et al., 2009). One report from 2020, showed that google searches for terms such as “sustainable activewear” increased by 151% percent with larger companies such as Adidas, Nike, and lululemon now featuring their environmental agendas and sustainability initiatives on their websites (Petter, 2020). There has also been an increase in smaller, more boutique activewear brands (such as Girlfriend Collective, Tala) which sell items made exclusively from recycled materials (i.e., plastic water bottles, bags) (Moon, 2017; Petter, 2020). While there is a growing environmental awareness within the activewear industry, much of this is concerned with the production and purchasing of clothing with little focus on the everyday practices of those who consume and use these products.

## Literature Review: Sport, Fitness, and the Environment

Since the late 1990s, scholars have increasingly explored the overlap between sport and the environment (Lenskyj, 1998; Wheeler and Nauright, 2006; Karamichas, 2013; McCullough and Kellison, 2017; Bunds and Casper, 2018; Wilson and Millington, 2020). In their review of sporting literature and the environment, Wilson and Millington (2017) divide this research into three broad themes; (a) sport management and environmental issues, (b) sport, modernization, and globalization, and (c) sport, neoliberalism and environmental policy. More recently, King and Weedon (2021) describe research focused on sport and the environment as emphasizing “the contradictions between sustainability rhetoric and capitalist growth,” with building critique of “‘ecological modernization' as an environmental strategy in the hosting of sport events” (pp. 1).

In line with Wilson and Millington (2017) and King and Weedon (2021), we acknowledge a large body of work interested in exploring the environmental effects of mega-sporting events and analyzing the sustainability policies of sporting organizing and leagues (McCullough and Kellison, 2017; Wilson and Millington, 2017). Scholarship has analyzed how mega sporting events negatively impact the environment because of increased tourism (i.e., more waste and transportation needs), an enormous carbon footprint, and long lasting negative environmental effects (i.e., infrastructure issues) (Collins et al., 2009; Ahmed and Pretorius, 2010; Hayes and Karamichas, 2012; Karamichas, 2013). Other scholars have been interested in sporting policy looking at the ways various sporting organizations (i.e., International Olympic Committee Agenda 2020 and Sustainability Strategy; United Nations Sports for Climate Change initiative) have developed (and often failed) to implement environmental sustainability policies (Trendafilova et al., 2013; Casper and Pfahl, 2015; Boykoff and Mascarenhas, 2016; Geeraert and Gauthier, 2018). While not focusing on large organization's sporting policies specifically, Mansfield (2009) explores the political ecology of the fitness industry's environmentalist agenda (encouraging outdoor exercise, emphasis on being one with nature, focus on sustainability and “clean living”). She illustrates how “fitness cultures are dominated by an environmental approach that tends to lead to green consumerism” (pp. 359) and in so doing, continues to prioritize human needs over environmental protection.

Another important thread in the sport and environment literature is the focus on action, lifestyle and outdoor sports, and participant relationships with the natural environment. Various scholars have explained how these often outdoor activities (i.e., surfing, climbing, skiing) facilitate intimate relationships with the natural environment (i.e., waves, mountains, rivers), which (in some cases) can lead to heightened “ecological sensibilities” (Olive, 2016) or “ecocentricity” (Brymer and Gray, 2010) [also see (Hill and Abbott, 2009; Humberstone, 2011; Stoddart, 2012; Wheaton, 2020)]. Some are extending such research by prioritizing Indigenous relationships with oceans, mountains, and other natural environments, and, at times, critiquing the longstanding and ongoing damage caused by settler colonialism and contemporary Western-derived recreation and tourism practices (Henhawk and Norman, 2019; Olive, 2019; Waiti and Awatere, 2019; Wheaton et al., 2019; Laurendeau, 2020; van Luijk et al., 2020). For example, Wheaton et al. (2019) look toward more Indigenous ways of understanding people's connections with “blue spaces” (oceans, lakes, coasts). Working with Māori ways of knowing well-being, they describe how an Indigenous viewpoint “affords alternative directions for research and reminds us that Eurocentric notions of what people do in oceans, rivers, lakes and on the shorelines are simply symptomatic of a particular world view and not of others.” (pp. 92). Continuing, they argue that it is time to anchor discussions around the environment and people to “different and more diverse ‘worlds' where research to date has been lacking” (pp. 92).

Similarly to Wheaton et al. (2019); Norman et al. (2020) suggest that one strategy to move beyond anthropocentricism and towards more-than-human ethics, especially regarding environmental change and environmental justice, is to look towards Indigenous ways of knowing that, although varied and diverse, “are holistic and inclusive of interconnective relations with more-than human worlds” (pp. 90). Through their research conducting sharing circles with elder members of the Fisher River Cree Nation (Canada), Norman et al. (2020) show how the elders' stories emphasized a deep, embodied connection with Land. These stories and ways of knowing “open up alternative ways of envisioning balanced and less anthropocentric relations between the planet, humans, sport, and physical activity” which have the “potential to foster a preferred environment and socially sustainable future” (pp. 98).

Research on sport and the environment is growing quickly, with scholars drawing from an array of fields (cultural studies, geography, political science, and urban studies) and exploring more diverse ways of knowing human relations with the environment. Various scholars have drawn upon Stuart Hall's “circuit of culture” and used a political ecological approach to trace the environmental impact and power inequalities created during the life-cycle of objects such as Nike's Air Jordans (Petrina, 2001) and surfboards and surfparks (Hill and Abbott, 2009). Other research has focused primarily on the development of sporting goods and the opportunities and challenges the sporting goods industry will face as they attempt more sustainable practices (Subic and Paterson, 2006; Subic et al., 2009, 2012; Subic, 2010). Subic and Paterson (2006) explain that as the sporting goods industry has adopted new materials and development processes to produce more advanced sports equipment, it has resulted in increased global environmental degradation. Therefore, Subic and Paterson (2006) argue that one way to address degradation is to develop more environmentally sound designs of sports equipment before production and to pay greater attention to the life cycle of products when developing them. Also, many environmental organizations have conducted research about the damaging effects of the production of textile and sporting goods (Dirty Laundry, 2011; Cobbing and Brodde, 2014). For example, in 2014, Greenpeace published a report about the environmentally harmful chemicals used in the production and dispersal of apparel and sporting goods from the 2014 FIFA World Cup (Men's Soccer) from major brands such as Adidas, Puma, and Nike (Cobbing and Brodde, 2014).

Regarding activewear more specifically, there has been limited scholarly engagement around the harmful effects of the production of activewear clothing on the environment. However, there has been research into the negative effects of the fast fashion industry and laundering practices of clothing that will be discussed throughout this paper (Anguelov, 2015). Importantly, there is a small body of literature that has explored the sustainability practices and policies of larger activewear and outdoor clothing companies (Horan, 2011; Ráthonyi and Ráthonyi-Odor, 2015; Erdnüß, 2016; Wang and Shen, 2017; Wu and Li, 2019). For example, Erdnüß (2016) explores Swedish outdoor clothing company, Haglöfs', sustainability initiatives showing that the brand's main motivation for sustainability is increased profit as the company believes there will be demand for environmentally consciously produced products in the near future. Similar to Erdnüß's (2016) findings, other scholars have been looking more closely at consumer behavior and the impact that a company's environmental sustainability policies and practices have on consumers' purchasing choices (Zheng and Chi, 2015; Nam et al., 2017; Ganak et al., 2019).

As mentioned earlier, many activewear companies are recognizing the important value consumers are placing on sustainability and environmentalism. In response, many have developed more initiatives and tactics to curb their environmental impact in addition to marketing strategies and campaigns to promote their environmental values (Petter, 2020). For example, the website of activewear juggernaut, lululemon, features a “sustainability and social impact” section outlining how their “products and actions avoid environmental harm and contribute to restoring a healthy planet” (LuLuLemon, 2018). Here they describe their sustainability efforts such as using renewable energy electricity in their facilities and using paper-based packaging rather than plastic, their use of more sustainable fabrics, and efforts to reduce waste. Other companies brand themselves as environmentally conscious and “eco-friendly” often using this “eco-identity” within their advertising and promotional strategies. For example, Patagonia, widely known for its environmental efforts, has a mission statement that reads, “We're in business to save our planet” (Patagonia.com, 2020). Patagonia and other companies also often emphasize the sustainability of the fabric (i.e., 100% recycled nylon, organic cotton, biodegradable merino wool) in their advertisements and clothing/product descriptions.

While many companies are trying to promote their ecological sensibilities, there have been a series of popular press articles, blogs, and websites that discuss the negative environmental impact of activewear (Dalton, 2017; Kay, 2017; Matera, 2019; Weekendbee, 2019). In a recent article in *Shape* fitness magazine, Falk (2020) outlines the devastating effect the apparel and activewear industry is having on the environment—i.e., use of non-renewable resources, production of greenhouse gases, production of carbon dioxide—and provides readers with tips as to how to purchase activewear sustainably. Similarly, there are many environmental groups (i.e., Greenpeace, World Wildlife Fund, Sierra Club) that have encouraged the public to understand how their daily actions (buying, washing, recycling) do have an impact on the environment. One popular organization is *The Story of Stuff Project*, inspired by Annie Leonard's popular animated short documentary of the same title, where she follows the lifecycle of material goods and works to expose the “the connections between a huge number of environmental and social issues, and calls us together to create an increasingly sustainable and just world” (The Story of Stuff Project, 2020). Larger environmental organizations also frequently post articles about the actions the public can take on a daily basis, which they argue can help slow environmental degradation. While there are many popular press sources that emphasize the “fine details” of everyday actions, there has yet to be any academic exploration into everyday usages of activewear and the connection between women's habitual fitness practices and the environment. In this paper we explain how our engagement with feminist new materialisms prompted us to rethink women's everyday practices, and to explore the sport-environment relationship as more-than-human phenomenon.

## Theoretical Framework: New Materialisms and Agential Realism

Referred to variously as the ontological or posthuman turn, vitalist theories, and “more-than-human” approaches, the new materialisms refer to an evolving scholarly tradition that challenges the anthropocentrism and logocentrism that has become dominate among the social sciences by having a concerted focus on the vitality of non-human matter (Coole and Frost, 2010; Dolphijn and van der Tuin, 2012; Fox and Alldred, 2018; Thorpe et al., 2020). In many new materialist approaches, matter is envisioned as “something more than ‘mere' matter: an excess, force, vitality, relationality or difference that renders matter active, self-creative, productive, unpredictable” (Coole and Frost, 2010, pp. 9). However, the focus is not only on matter, but the ways the human and non-human relate and are connected. There is a concerted effort to “situate citizens, ideas and values (as well as theorists themselves) within the fields of material forces and power relations that produce and circumscribe their existence and co-existence” (Coole and Frost, 2010, pp. 28). This emerging body of knowledge is informed by an eclectic array of disciplinary foundations and draws upon the work of contemporary thinkers such as Karen Barad, Rosi Braidotti, Jane Bennett, Gilles Deleuze (with Felix Guattari), Donna Haraway, Bruno Latour, and Brian Massumi, among others. Over the past decade, a series of comprehensive volumes espousing the promise of new materialist thought have chronicled its emergence, contributions, and underlying tenets (see Alaimo and Hekman, 2008; Fox and Alldred, 2018; Thorpe et al., 2020).

Importantly, many have noted the similarities between new materialist thought and Indigenous knowledges, cautioning new materialist scholars against “failing to acknowledge and seriously engage the Indigenous scholars already working with parallel concepts” which risks “practices of erasure of Indigenous cultures and thought” (Dolphijn and van der Tuin, 2012 pp. 2; Watts, 2013; Todd, 2016; Ravenscroft, 2018; Clary-Lemon, 2019; Rosiek et al., 2019). Whereas some reject new materialisms outright for these failings, others encourage a more collaborative and diffractive approach between new materialist literature and Indigenous ways of knowing where these bodies of literature “inform one another, extend their respective influence, and bring what benefits are latent within them to local and global communities” (Rosiek et al., 2019, pp. 2). Addressing these critiques and the possibilities of collaboration, there is small but growing number of scholars who are working at this intersection of Indigenous knowledges and new materialisms to think about environmental concerns in the 21st century (Thomas, 2015; Yates et al., 2017; Celermajer et al., 2020). Working in the context of Aotearoa New Zealand, Thomas (2015) advocates for Indigenous more-than-human (MTH) approaches that examine the “considerable synergies” between more-than-human theorists and Indigenous understandings of non-human agency and kinship. She examines how Ngai Tahu (a Māori tribe) advocated for the Hurunui River as lively and agentic, thus successfully expanding their catchment to include the river. In her observations of this process, she concludes that “there are generative possibilities for MTH theorists to work alongside Indigenous communities and carve political space for more people to advocate for a relational ethics” (Thomas, 2015, pp. 974).

Recently there has been an array of scholars exploring the possibilities of using new materialisms and post-humanist or more-than-human theoretical approaches to build upon and extend previous ways of knowing the environmen (Alaimo, 2010, 2016; Pierides and Woodman, 2012; Schmidt, 2013; Gough, 2016; Neimanis, 2016; Bastian et al., 2017; Fox and Alldred, 2020a,b). One notable piece is from Hird (2012) who uses feminist science studies (FSS), and engages with new materialist scholars (Haraway, Barad), to study waste as a means of contributing to discussions and literature on feminist epistemological concerns around the inhuman. She describes the ephemerality of knowing waste and how waste (socially, materially, ideologically) evades definition through current humanist lenses. For example, she describes how within many contemporary societies, “waste” is an “ambitious linguistic signifier” (pp. 454) where anything and everything can be understood as waste depending on context and the perceiver. Boundaries around “waste” are indefinable. Within engineering and waste management control, the indeterminacy and liveliness of the landfill assemblage (the various unique intra-actions that occur in individual landfills of bacteria, waste, sunlight, temperature) means containment of waste ultimately fails. Hird argues that these attempts to contain waste fail because in these attempts, waste is viewed through an anthropocentric lens, seen as an inert, passive entity acted upon by humans. Therefore, she conceptualizes issues with waste as a “problem of inhuman knowing,” and in so doing, “brings to the fore the inherent indeterminacy of the world rendered determinate, by human and inhuman alike” (pp. 465).

Increasingly, some sporting scholars have begun to engage with new materialist and posthumanist thinking to (re)envision the relationship between physical culture and the environment (Millington and Wilson, 2017; Evers, 2019a; Thorpe et al., 2020, 2021). Building upon and extending the strong theme in sport and environment literature, some scholars have used new materialist theory to rethink mega-sporting events. For example, McDonald and Sterling (2020) apply a new materialist, Baradian lens to rethink the ideas around athletes, the environment, and the polluted, “troubled” waterways (beaches, lagoons, bays) of the 2016 Rio de Janeiro Olympic games in Brazil. Rather than focusing on the impact of polluted waterways on athletes and the games, McDonald and Sterling (2020) explore the polluted waters of the games as “an ongoing, interconnected, ethical, and onto-epistemological process,” within which “human bodies are entangled with multiple non- and more-than human bodies” (pp. 296–297). In a similar vein to Millington and Wilson (2017); McDonald and Sterling (2020) discuss the development of the Trump Golf Course in Scotland with a focus on sand dunes. Challenging the anthropocentrism of sporting practices and much critical sport scholarship, they ask readers to consider sand dunes as active players alongside people such as Donald Trump and sporting organizations.

Other sporting scholars have explored action sports and the environment using a posthumanist, new materialist lens. Through films, art exhibitions, journal articles, book chapters, and online blogs, Evers (2019a,b) has written about the connection between surfing, the environment, and new materialisms. He emphasizes the need to take a post-humanist, material-social approach to recognize how humans intra-act with more-than-human worlds—pollution, capitalism, and environmental crises. Similarly, Booth (2020) uses the case study of Bondi Beach to explore the agentic qualities of beach and environmental matter regarding sunbathing and surfing. In so doing, he shows how “geomatter acts directly on, and inscribes itself in, corporeal matter to produce effects and sensations” (pp. 250). A recent special issue on blue spaces, sport, and well-being featuring papers on ocean swimming, surfing, sailing/yachting and *waka ama* paddling, offers another important contribution in exploring the human and more-than-human relationships to seas and oceans (see also Olive and Wheaton, 2021).

Also within the sporting scholarship, scholars have focused on non-human matter as a way to think differently about the impact of sporting participation and consumption practices on animals (Atkinson, 2014; King, 2020). In so doing, some sport scholars are engaging with inter- and multi-species approaches to explore human-animal relationships in sport, including horses (Dashper, 2019) and canines (Merchant, 2020). A particularly noteworthy example is King's (2020) new materialist, post-humanist exploration into the environmental impact of the increased whey protein powder consumption by fitness enthusiasts (see also King and Weedon, 2020). Beginning with an understanding of the animal protein as a “dynamic and lively material-discursive subject” (pp. 202), King (2020) became increasingly interested in what it “does to humans and other life forms as it circulates in and through diffuse assemblage of bodies and environments.” (pp. 202) Building from this research, King and Weedon (2021) recently introduced the concept of “ecological embodiment” as a way to think about body-environment relations in less dualistic terms. They argue that “ecological embodiment” can be understood as a “fluid bodily state of becoming” (pp. 2) and as a “sensibility for thinking about the intra-active (Barad, 2007) relations among humans embodied practices and the rest of what gets called ‘nature'” (pp. 2). Taking inspiration from King (2020), King and Weedon (2020), and other scholars, here we explore the everyday, “mundane,” and the often overlooked, body-environment intra-actions that are part of (women's) habitual fitness practices. In so doing, we engage specifically with Karen Barad's theory of agential realism.

### Agential Realism

In this paper, we explain how our engagement with feminist new materialist scholar Karen Barad's theory of agential realism prompted us to rethink the more-than-human relations with fitness practices, moving bodies, clothing, and the environment. Barad's work is featured across various new materialist scholarship and has been described as “one of the most influential and important representatives of contemporary materialist scholarship” (Lemke, 2014, pp. 5). A unique element of Barad (2007) theorizing is her ability to bring quantum physics and the social sciences into dialogue, often drawing upon physics principles to articulate her central onto-epistemological concerns. Her seminal theory of agential realism is inspired by the work of physicist Niels Bohr who questioned the belief in the existence of independent autonomous entities and emphasized the impactful role of the measuring apparatus. Bohr argued that there is no independent reality with well-defined properties that can be measured, and instead showed that the properties realized in an experiment are dependent on the measuring apparatus (Pinch, 2011). Therefore, for both Bohr and Barad, the primary ontological unit is not an independent object, but a phenomenon defined as “the ontological inseparability of agentially intra-acting components” (Barad, 2003, pp. 815). Extending Bohr's understandings, however, Barad brings quantum physics into conversation with ideas from critical social theory (i.e., Foucault) and particularly feminist post-structuralism.

Importantly, Barad's concept of intra-action is ontologically distinct from “interaction” which assumes two distinct boundary-ridden entities relating to each other. In contrast, intra-action insists upon the inseparability of those entities. Højgaard and Søndergaard (2011) elaborate on this by explaining: “The concept of intra-action demands a thorough co-constitutional thinking … It is the co-constitution—the intra-action of subject and object—that forms the subject matter (so to speak) of the analysis” (pp. 347). Within phenomena, there are many intra-actions that occur resulting in an entanglement of various entities/things/objects. For Barad, to be entangled “is not simply to be intertwined with another, as in the joining of separate entities, but to lack an independent, self-contained existence” (Barad, 2007 pp. ix).

Central to Barad's theory is the understanding that these intra-actions and entanglements are productive, performative, and agentic in the world's becoming. Within agential realism, Barad stresses that agency is not something that can be possessed or correlated with intentionality. Believing that a person or object can exert agency “pulls us back into the same old humanist orbits over and over again” (Barad in interview with Dolphijn and van der Tuin, 2012, pp. 54) which new materialisms and agential realism strive to move away from. Instead, Barad (2003) defines agency as “‘doing'/‘being' in its intra-activity. Agency is the enactment of iterative changes to particular practices through the dynamics of intra-activity” (pp. 827). Thus, for Barad, agency is about dynamism, it is an ongoing relational process that focuses on the energy and vitality created through material-discursive intra-actions.

This understanding of agency moves the focus away from exploring only human or non-human actions, and reorients thinking toward the way humans and non-humans (i.e., waste, clothing, water, dirt) are always entangled, and how such entanglements are productive in that they “do” something. Humans do not act in isolation, but are always intra-acting with non-human matter and therefore, scholars must “take matter seriously” (Hein, 2016). When thinking about sport and the environment, a Baradian approach encourages a movement away from the anthropocentric lens that focuses primarily on human activities, and instead recognizes “ecologies as socio-natural lifeworlds through which bodies are materially co-constituted, and bodies as ecologies composed through dynamic, uneven, multispecies, and multiorganismic entanglements” (King and Weedon, 2021, pp. 6).

## Methodology and Analysis: Toward More-Than-Human Understandings of Athleisure

As discussed above, broadly, new materialisms and agential realism seek to de-center the human and acknowledge the distribution of agency among material and discursive forces. This ontological approach has resulted in some scholars identifying a dissonance between new materialisms and the familiar qualitative methodologies that emerged through the interpretivist and constructivist paradigms with roots strongly in humanism. In response, some have suggested that humanist qualitative research in which data collection, analysis, and representation typically rely heavily on language and human experience, “need to be reequipped with different tools” (Markula, 2019, pp. 6) resulting in various scholars proposing methodologies and methods that are more in line with new materialisms' ontology (St. Pierre, 2011; Fox and Alldred, 2015; Koro-Ljungberg, 2015; Nordstrom, 2018; Markula, 2019). One such approach is Karen Barad's theory-method concept of diffraction.

### Diffraction

In an interview with Juelskjær and Schwennesen (2012), Barad discusses that her theorizing was developed by reading “all different kinds of things from different fields at once; physics, philosophy, science studies, feminist and queer theories” (pp. 11). Within agential realism, she brought together the philosophy of physics of Niels Bohr with writings from feminist and queer scholars, in particular drawing upon Donna Haraway, Judith Butler and Michel Foucault. Throughout agential realism, Barad emphasizes the need to bring ideas from different disciplines together and to read insights from varying fields together and through each other. She describes this practice as diffraction, an approach which places “understandings that are generated from different (inter)disciplinary practices in conversation with one another” (Barad, 2007, pp. 92–93).

Diffraction, as a form of methodology and analysis, uses the physics concept as metaphor. Within physics, diffraction is defined as the “way waves combine when they overlap and the apparent bending and spreading out of waves when they encounter an obstruction” (Barad, 2007, pp. 28). McKnight (2016) poetically links the physics phenomenon to methodology, describing how diffraction encourages a “reading [of] approaches through each other, as waves pass through the narrows of a rocky outlet, and are transformed, heading in different directions, making new patterns” (pp. 197). Barad emphasizes that diffraction encourages reading different approaches through each other, such that scholars must be attentive to the “fine details,” the everyday practices and intra-actions that are often overlooked and taken-for-granted. However, it is not simply approaches and ideas from different disciplines that can be “read together,” but also different “forms of data.” In her new materialist inspired research on the intersex phenomenon, Linghede (2018) understands diffraction as a “process of ‘reading-the-data-while-thinking-the-theory'” that works to “disrupt thought as we plug in multiple texts and concept into data and read them through one another” (pp. 574). For our project, diffraction resulted in bringing together interview data, with our own experiences with activewear, with data from the larger project, reading these together and through a range of literature from environmental studies and with new materialist theory.

Within the broader project from which this paper derives, we used a variety of qualitative methods (i.e., interviews, focus groups) with more experimental, creative methods (i.e., moving methods, digital diaries, art-based methods working with the materials of athleisure). Interviews and focus groups were conducted with women aged 18–65 living in Aotearoa New Zealand who owned at least five pieces of activewear clothing (defined on recruitment materials as casual clothing designed for physical activity). In total, there were five focus groups consisting of between 5 and 10 participants each, totaling 35 participants. The focus groups were designed to contextualize understandings of activewear in Aotearoa New Zealand, as well as have participants become familiar with the project. After the focus groups, participants were invited to complete a 2 week photo diary taking pictures of either themselves in activewear or the activewear itself in the various places it “lives” (i.e., gym bags, drawers, bedroom floors, washing lines). The women who completed the photo diary then participated in a semi-structured interview lasting between 45 minutes and an hour and a half. In total, 22 women from the focus groups completed a photo diary and interview.

We are aware that some post-humanist scholars have critiqued the use of interviews (and other humanist-based methods) as ontologically incongruent with new materialisms (St. Pierre, 2011), and we have recognized these tensions and debates elsewhere (Brice et al., 2020; Thorpe et al., 2020). The first author conducted semi-structured interviews with an interview guide created and developed using new materialist and agential realism concepts, using questions that focused more on human-activewear entanglements and intra-actions than human experience and emotions alone. As discussed earlier, the broader research project was not developed with the explicit aim to explore the environmental impact of activewear or fitness practices, but was rather about the possibilities of using new materialisms, and agential realism more specifically, to explore the activewear phenomenon. Therefore, the interview questions were not primarily based around women's environmental practices, but activewear more broadly (i.e., why they bought certain clothes, their relationship with clothing). Within these interviews, a small sub-section of questions focused on women's washing practices and their consumption behavior. In addition to the interviews, we conducted various creative methods such as having participants come in for another focus group where they cut apart old activewear and then used the materials to create something anew. Simultaneously, we embarked on our own year long collaborative experimental project where we “lived with the sports bra” (Brice et al., 2020). While these are the “methods” we used, it was the diffractive process—reading insights from the data, through our own experiences, and always with the theory—that prompted us to new noticings in the human-environment intra-actions in the activewear phenomenon.

Diffraction is a process of knowing, an entangled engagement “as part of the world in its differential becoming” (Barad, 2007, pp. 88). It is not an autonomous human researcher looking at data collected from various research methods, but rather the process is understood as comprised of unfolding, performative, intra-actions involving humans, non-humans, discourses, time, and space. Barad (2007) writes that “knowing does not come from standing at a distance and representing but rather a direct material engagement with the world” (pp. 49). Therefore, the researcher is understood as entangled with the research process. It was our own entangled experiences of wearing and living with activewear while engaging with Baradian theory, and reading such experiences diffractively through interview data, that we first came to new noticings about the relationship between activewear and the environment. According to MacLure (2013a,b), there are occasions in the research process when something (phrases in an interview, fragments of field notes, an object) begin to “glow,” such that it “seems to reach out from the inert corpus (corpse) of the data, to grasp us…they exert a kind of fascination, and have capacity to animate further thought.” (MacLure, 2013b, pp. 228). As we read through the various data, the laundering and the environmental entanglements of activewear started to “glow.” From here, we engaged more deeply with new materialist theory and environmental literature to pay more attention to the complex human and more-than-human relations involved in the athleisure-environment phenomenon. In this way, our analysis did not involve coding as would be typical of a more conventional qualitative analysis. Instead, working within new materialisms, we understand analysis as being a diffractive process occurring throughout the entire research process itself. This process led to a range of new understandings and ideas regarding the relationship between activewear, society, and the environment (for examples, see Brice et al., 2020; Brice and Thorpe, 2021b).

## Activewear-Environmental Entanglements

In the remainder of this paper we focus on two activewear-environmental entanglements to reveal some of the many activewear and fitness intra-actions with the environment. The first explores the connection between sweaty activewear and the production of microplastics that end up in the ocean. The following looks at secondhand activewear and the increase of waste in landfills. Both of these examples begin by describing some of the anthropocentric discourses around sweaty activewear and “old” clothing, followed by a discussion of how these discourses materialize through various intra-actions of activewear with other non-human elements.

### “I Do the Sniff Test … and Know They Really Need to Be Washed”: Laundering Activewear

In Atkinson (2017) ethnographic research on the sensuality of sweat, he writes that “there can be no doubt that in most cultural contexts, sweat is a stigma. Sweat smells are unpleasant; sweat uncomfortably sticks skin to clothing, and leaves a bacteriological trace” (pp. 64). This “sweat stigma” was discussed in many of our interviews with participants. The women often talked about the efforts they take to reduce, what Hannah^3^ (50 year old, active exerciser) described as, the “sour smell” of activewear clothing because they wanted to avoid being embarrassed at the gym by their body odor. One of the common tactics to avoid such stigma was to wash activewear immediately after wearing:

Years ago I used to do that—let it dry out a little bit and wear it again— but then it gets that sour smell. I've been besides someone on the treadmill with a sour smell and it's really not nice. I know what it is now but back then I didn't realize it was a sour smell until I smelled it and said “oh my goodness, it's dry sweat. That's *disgusting*” (Hannah, emphasis added).

This social stigma around sweaty smelling activewear resulted in her washing her clothes everyday: “Now everyday they are washed … I soak them in water because of the sweat.” Similarly, Kae (52 year old, highly active exerciser) discussed her routine for her sweaty clothes, saying: “After [workout], I actually get undressed in the shower and rinse them right away and then put it in a towel. As soon as I get home, I empty the bag into the washing machine and do that load.” Once in a while, Kae would let her sports bra air dry and then, according to her, “I usually do the sniff test and know, ‘Oh, I've worn those a few times, they really need to be washed'”.

The desire to wash activewear after every (or every other) use was not related to the functionality of the clothing or health factors, but was primarily because of the fear of the sour smell, or rather the fear of social stigma associated with this smell in public places. According to various dermatologists, there is no serious health risk to re-wearing unwashed activewear after one or two uses. Some doctors have noted that unwashed activewear has a higher concentration of bacteria than clean activewear which can lead to acne and skin irritation (at worst, yeast infections) for some people, but it is not very common (Malacoff, 2017; McCoy, 2019). Instead, dermatologists and popular press articles point more toward the body odor smell that will occur when re-wearing activewear. As our interviews revealed, many exercising women have come to recognize the smell of sweaty activewear as unpleasant and therefore, wash their clothes on a daily basis to avoid the negative social consequence of being the “smelly one.”

**Figure 1:**
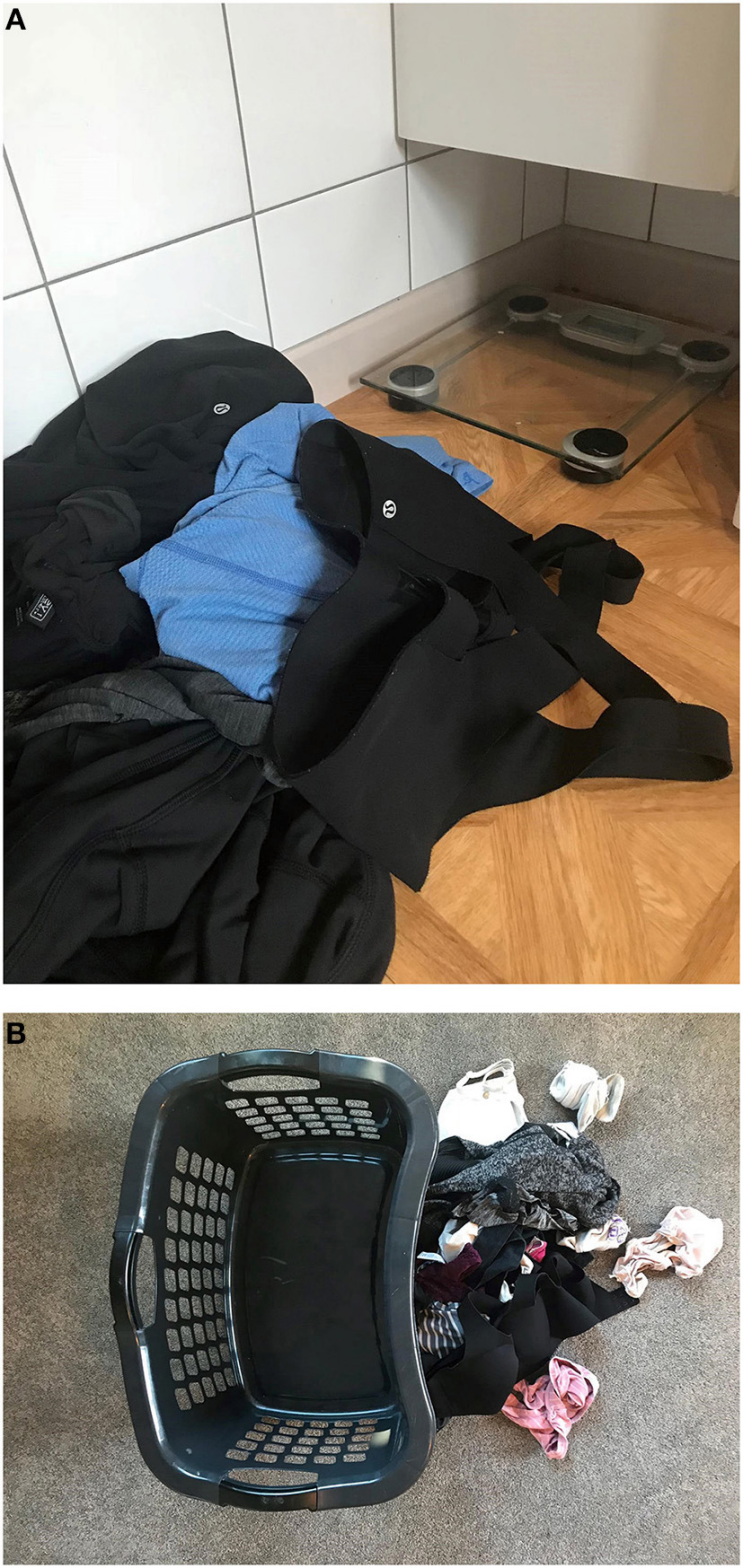
**(A,B)** Images of activewear in laundry piles collected during our “living with the sports bra” experiment.

While there is not a serious health risk when rewearing unwashed activewear, being the “smelly, sweaty one” does stand in contrast to socially acceptable notions of “fit femininity.” Scholars have described how, in many cultures, feminine respectability “consists in conforming to norms that repress sexuality, bodily functions [i.e., visible sweat], and emotional expression” (Waitt and Stanes, 2015, pp. 136). Having a sweat free, non-smelly, “clean” and controlled body becomes a key element of respectable femininity and moral cleanliness (Young, 1990; Shove, 2003). Classen et al. (1994) elaborate that in Western conventions, “while men are allowed to smell sweaty and unpleasant without losing any of their masculine identity, women who don't smell sweet are traitors to the ideal of femininity and objects of disgust” (pp. 164). Women wash their clothing not only because of broad social discourses around odor, but also in response to the gendered politics of smell and acceptable femininity. Through our “living with” the sports bra experiment (Brice et al., 2020), we came to question such gendered norms and their environmental impact, as seen in the following comments: “Today I grabbed my sports bra from the dirty pile on the floor in my bathroom and wore it to my workout. It really wasn't the end of the world to wear a slightly damp (not too stinky) sports bra in public,” “A sweaty sports bra will be the least of our worries when our children are witnessing the tragedies of environmental devastation,” and “Hey, maybe there is a feminist environmental politics in this: wear your sports bra more than once before washing and help save the world, one less wash at a time. The politics of sweat-smell in spaces of fitness!” (Figures 1A,B).

Barad's (2007) agential realism encourages scholars to explore how these discourses around body odor and “fit femininity” “are always already material” (pp. 152), and how the “material and discursive are mutually implicated in the dynamics of intra-activity” (pp. 152). Therefore, there is an emphasis on how these discursively produced ideas materialize and are entangled with non-human matter and ecologies. When laundered, activewear (and its particles) is intra-acting with other non-human matter (e.g., water, detergents, waterways), becoming part of new entanglements that are agentic and powerful, negatively impacting the environment. Every time activewear is washed, it intra-acts with laundry detergent and water molecules that then get released into water processing plants. Activewear clothing is made from a range of different materials often comprising of a mix between synthetic (human made) and organic depending on the support needed, sweat-wicking properties, and breathability (ÖzdIl and Anand, 2014; Josephson, 2015). Research has shown that the synthetic materials within activewear (and other synthetic/mixed material clothing) shed microfibers (a type of microplastic) into the laundry water every time they are washed (O'Loughlin, 2018; Ross, 2019; Herweyers et al., 2020; Yan et al., 2020). Once laundered, the activewear microplastic-water is treated at wastewater treatment plants (WWTP). Advanced WWTPs can remove up to 90% of microplastics from soiled water. However, many WWTPs across developed countries have less efficient technologies and thus can only remove an average of 50%, while many developing countries do not have the capability to treat wastewater at all (Herweyers et al., 2020). After moving through WWTPs, the activewear-microplastics then travel to aquatic features across the world (streams, oceans, lakes, rivers). One study suggested that on average, 34% of all microfibers from laundering end up in the ocean (Mishra and Rath, 2019). According to non-profit organization Ocean Clean Wash, plastic particles washed from synthetic clothing contribute up to 35% of the plastics in the world's oceans (Ocean Clean Wash, 2019). Interestingly, a recent study shows that activewear clothing does not release a greater amount of microfibers than other forms of clothing, but due to the greater frequency of washing, it does have a greater overall impact on the increased prevalence of microplastics in the environment (O'Loughlin, 2018).

Unlike larger pieces of plastic (straws, fishing nets, bottles) that may be more readily detectable and have been part of numerous environmental campaigns (cleaning up the beach day, reducing plastic straw usage), microplastics are so small they are not visible to the human naked eye and are therefore harder to remove from the ocean. This has resulted in microplastics being found in the bellies of baby fish, shellfish, crabs, and even found in tap water, beer, and sea salt samples as water is taken from the ocean and treated for human consumption (Kosuth et al., 2018; Ross, 2019). One sample in Brazil found that 83% of all fish caught contained microfibers, with another study suggesting that 1.4 trillion microfiber particles are present in oceans, which has serious consequences for marine life such as starvation and reproductive issues in fish (Mishra and Rath, 2019). The microplastics scholarship is a relatively new field, and therefore, the research into the effect of microplastic consumption on human's health is an emerging and developing topic. However, recent scholarship has suggested microplastics in the human body will have negative effects on human health and can result in chronic inflammation that increases the risk of cancer and increased risk of neoplasia (uncontrolled growth of cells resulting in tumors or mass lesions) (Wright and Kelly, 2017; De-la-Torre, 2020; Prata et al., 2020).

While the washing and laundering practices of activewear are often far removed from these broader ecological systems, agential realism encourages a recognition of the ways both humans and non-human matter become part of, and are connected to, many other entanglements. Often concerns regarding sweaty activewear are only viewed in relation to the harm it may cause humans (social stigma, skin irritation). Such anthropocentric ways of thinking can lead to excessive laundering of activewear and an oversight of the ways sweaty activewear is entangled with many other ecological forces and entities. However, Baradian theory encourages a recognition of matter as an “active participant in the world's becoming, in its ongoing ‘intra-activity',” such that such that we must better “understand how matter matters” (Barad, 2012, pp. 803). When washed, activewear *does* something, it intra-acts with the water, the microfibers breaking down, and in so doing, is impactful. Sweaty activewear “matters” not only in its relation to humans, but in its connectivity with the environment and other non-human entities.

Various environmentalists, environmental groups, and popular press articles have emphasized the negative impact of washing practices on the environment (Brian, 2019; Ocean Clean Wash, 2019; Petter, 2020). For example, in an article in *The Guardian* discussing the carbon footprint of laundry, the authors write, “no one wants to go around smelly, but it's worth at least asking the question: does stuff go into the wash unnecessarily often?” (Berners-Lee and Clark, 2010). With growing awareness of the impact of laundering practices, such discussions often emphasize the connectivity of humans with nature and the importance of small actions to help reduce environmental degradation. Yet, this approach is rarely applied within sport sociology literature to explore how the micro-actions and non-human matter within sporting cultures are entangled with the environment. A new materialist, Baradian approach, however, begins from an onto-epistemology of relationality where humans are fundamentally seen as inseparable from their environments, therefore providing different perspectives and ways of studying sporting cultures and the environment.

### What It Really Means to “Biff It Out”: Activewear and Landfills

Throughout this project, the ethics of when and how to discard activewear continued to emerge. Bringing together the interviews, photos, our experiences, and data from other methods, it became even more evident that the large amounts of activewear being consumed and discarded by millions of women around the world are having a significant environmental impact. Speaking with participants, many of the women discussed their concern for the environment and efforts they take to reduce their environmental footprint. For example, Janice (a 25 year old Crossfit exerciser) discussed how she often upcycled her old activewear clothing turning them into pillows or dog beds. Sherry (a 24 year old yoga participant) described how she bought secondhand activewear primarily because she wanted to reduce waste. She discussed buying secondhand lululemon leggings from an internet auction site (Trademe) because “I could get the quality, the sustainability, plus it fit my values more, again, that I wasn't buying it from the shop, I wasn't making the shop produce more. I was buying it secondhand so saving it from being wasted.”

**Figure 2:**
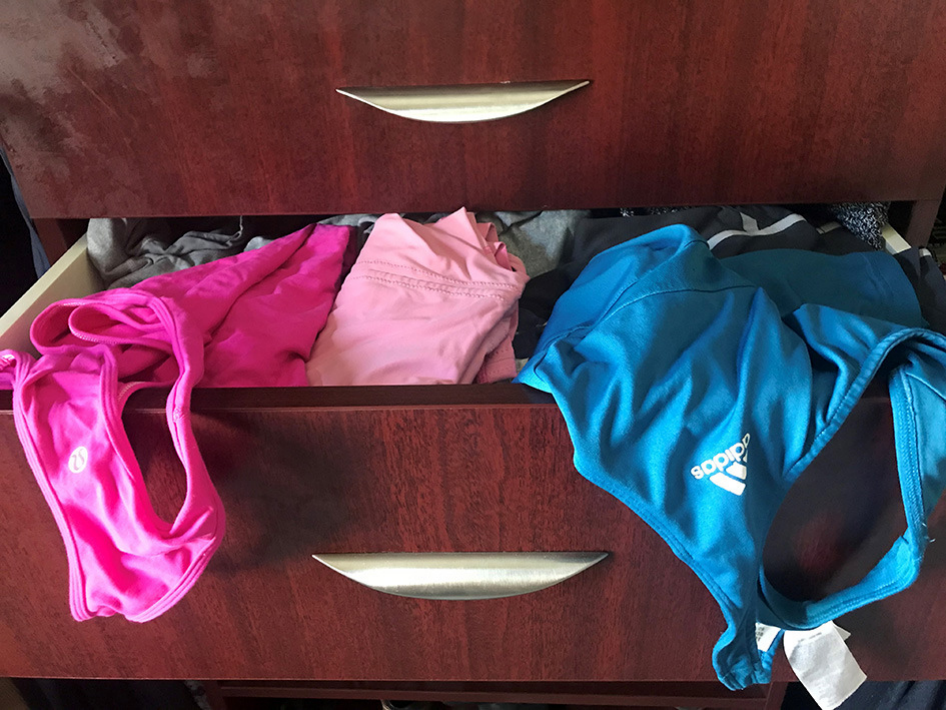
An image of an overflowing activewear drawer.

While many of the participants did discuss the efforts they take to recycle their clothing and to minimize their purchasing of activewear, in Aotearoa New Zealand (as in many Western cultures) there is a culture of fast fashion and conspicuous consumerism. In part because of neoliberalism and capitalism, many cultures have shifted to consumption practices where one's happiness, identity formation, and status in society is linked with material possessions (Hamilton, 2010). As Dittmar (2011) describes, “having the “right” material goods has become vital to many, not so much because of these goods themselves, but because of hoped-for psychological benefits, such as moving closer to an ideal identity, creating a desired social image, and achieving positive emotional states” (pp. 745–746). Within the 21st century, where wellness and health are often prioritized as a result of neoliberal and healthism discourses, purchasing and wearing the latest activewear designs is one way that women can present themselves as conforming neoliberal citizens (Brice and Thorpe, 2021a). In other words, by wearing activewear in public spaces, women are (subconsciously) performing an “intent” to exercise (whether they do or not is much less relevant) and thus may be read as “good” neoliberal citizens, proactively managing their own health through an active lifestyle. Activewear also becomes linked to femininity as it has become “a visual image of fashionable female modernity” that works to intensify the “symbolic value of an active body” (Horton et al., 2016, pp. 191). Through an entanglement of consumer culture, neoliberalism, capitalism, and healthism, the buying of activewear becomes a (not unproblematic) part of the “fit femininity” phenomena (Figure 2).

**Figure 3:**
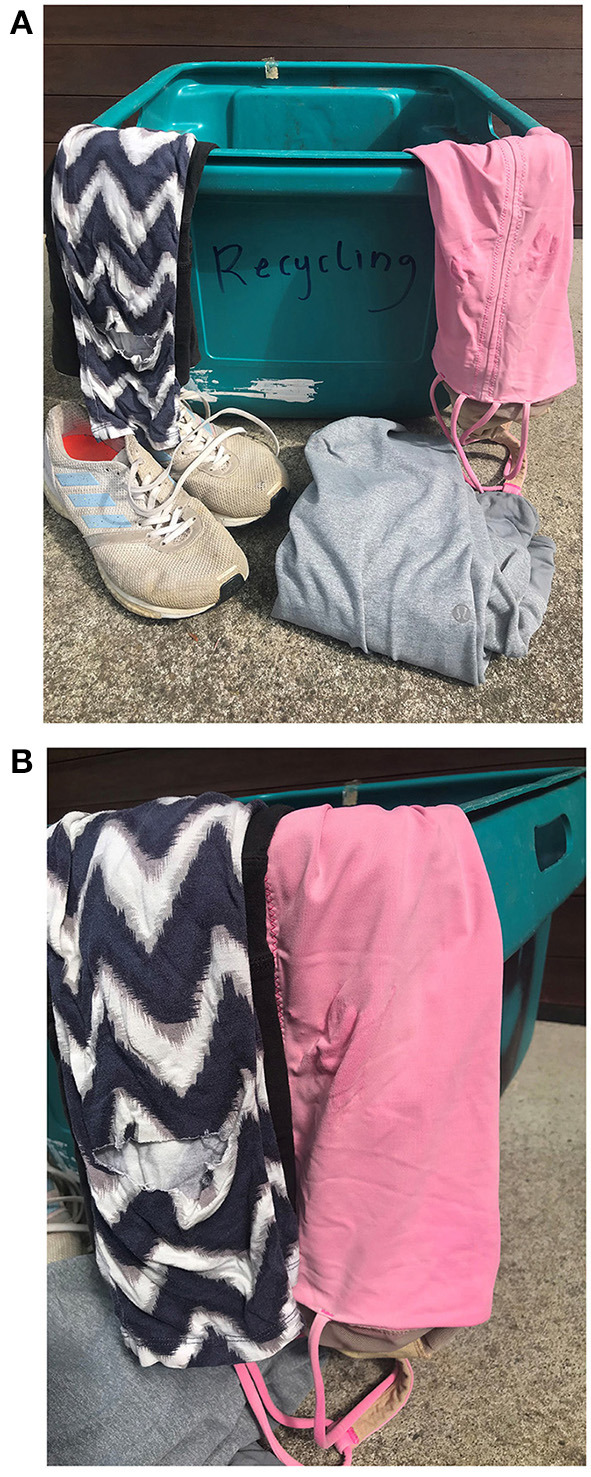
**(A,B)** Images of discarded activewear with holes, faded colors, and pulls.

The continuous purchasing of activewear meant that many of our participants found themselves with dressers overflowing with activewear. Such revelations prompted us to ask them about the “retiring of their activewear” (i.e., “When do you “retire” your activewear? Where does it go?”). Many women responded that they “get rid of them” (Georgia, a 55 year old recreational exerciser) or “biff them out” (Caroline, a 31 year old amateur boxer) with little consideration for the environmental impact of such conspicuous consumption and disposal practices. Others gave more thought to the after-life of their activewear garments, opting instead to donate their used items to family members, friends, or to opportunity shops. These decisions to “retire” the clothing were based primarily around the importance of the activewear to the human; it no longer supported the body, the color had faded or gone out of fashion, there were holes in the fabric. While these are all common and acceptable excuses in modern Westerns societies, they do come from an anthropocentric view where human interests are prioritized over the non-human. Once activewear leaves the human (“biffed out”), it is no longer given any thought; where it goes, what it does, who/what it interacts with along the way, are of very little importance (Figures 3A,B).

In her political ecological analysis of “waste,” Hird (2012) describes how waste is an “ironic testimonial to a desire to forget” (pp. 455). Once garbage is placed into a receptacle, it is quickly forgotten and rarely talked or thought about. In this way, she writes, “landfills make their appearance on and in the landscape as a material enactment of forgetting” (pp. 455). In our research, even for those participants who were environmentally conscious and did not want to contribute to growing landfills, their primary concern was for activewear immediately after it left them with less concern about what happens once in the landfill. There was less acknowledgment of the way activewear becomes part of other non-human entanglements and part of agential intra-actions that are impactful. Much like the waste in Hird's study, once the object of activewear leaves the human owner, it is forgotten about. However, an agential realist account encourages a move beyond anthropocentric “forgetting,” instead giving “matter its dues as an active participant in the world's becoming” (King, 2020, pp. 136). Therefore, similar to the laundering practices, when engaging with Baradian theory we were encouraged to look more carefully at how the activewear continues to “live on” long after it leaves the human “owner” of the fitness objects.

During one of the creative methods where we cut apart activewear clothing, the participants talked about how actually cutting apart the clothing brought into realization the negative environmental impact of their consumption and disposing practices: “I think the other thing that is bad is … clothing is one of the worst things, manufacturing of clothing is one of the worst things for the environment,” “I didn't feel guilty … then I realized this was a really good item and should have given it to an op[portunity] shop or something cause someone could've used it,” and “[Holding up an old item of activewear] this is going to landfill. That has suddenly made me feel really bad … That's been a big moment for me.” By engaging with the materiality of the clothing itself and thinking about its various human and more-than-human entanglements, there began to be a more conscious awareness of the way clothing will continue to be affective after it leaves the women.

Research indicates that because of the very nature of activewear (used for physical activity, supports the body, high rate of laundering), it has a shorter product life and high disposal rates (Subic et al., 2012). Therefore, much of the activewear purchased becomes part of the landfill system. In recent years, the amount of textile waste in landfills has been drastically increasing. One study suggests that 75% of the textiles manufactured each year is sent to landfills by consumers (Textiles Timaru, 2015). In 2015, over 10 million pounds of textiles entered landfill in the United States, while only 2 million were recycled (made into post-consumer goods) (United States Environmental Protection Agency, 2017; Dohms, 2019). Although not at the same scale as the United States, in Aotearoa New Zealand textiles are becoming the fastest growing source of waste in landfills. Many scholars credit the increase in fast fashion, the short lifespan of much contemporary clothing (including activewear), and the materialistic consumption culture of many Western countries, for the high rates of textile disposal and waste in landfills (Subic et al., 2012; Woolf, 2019). The activewear discarded by fitness consumers becomes part of this landfill entanglement, an intertwining of materiality, waste, Western human cultural practices of consumption, the desire for new and “clean” activewear, and government protocols around disposal.

Once in a landfill, the object of activewear (i.e., lycra pants, sports bra) intra-acts with other waste, heat, moisture, weather, and microbes. Together, these entities are mutually constituted within an entanglement that is agentic, contributing to the production and accumulation of greenhouse gases. Karanjekar et al. (2015) describe how waste degradation and waste composition changes dramatically within landfills based upon the waste contents, moisture, ambient temperature, pH, and rainfall, all of which affect the amount of methane gas that is produced and released from the landfill. While methane gas can be collected for energy production, it is also released into the atmosphere contributing to a rising global temperature and climate change. One popular press article cited that every kilogram of clothing in landfills creates 3.5 kilos of greenhouses gases resulting in the fashion industry creating roughly 10% of the global carbon dioxide emissions (more than aviation and shipping combined) (Woolf, 2019). Although, the discarded object of activewear is no longer directly intra-acting with the human owner of the object, it is still a powerful entity, becoming part of other entanglements, and through these entanglements, activewear “does” something that few consumers consider in their purchasing and disposal decisions. In this case, it is the increased production of greenhouse gases that contributes to climate change and environmental degradation.

Climate change in itself is entangled with systems of oppression and gender. As feminist environmental justice scholars reiterate, it is women from poorer countries who will be (and are being) most negatively affected by the changing environmental landscape (Denton, 2002; Arora-Jonsson, 2011; Norgaard, 2012). The reasons for this are many, but one report by the Global Citizen organization discusses how women are more at risk because they tend to live in higher poverty than men (70% of the world's poverty population are women), they have less access to human rights to be able to move freely and acquire land, and face systemic and structural violence (McCarthy, 2020). These vulnerabilities to climate change are a result of “a history of colonization, global capitalism, and regional geophysical determinants [that] direct flows of power and maintain important differences between communities while difference of age, gender, race and ability/mobility are similarly salient” (Neimanis et al., 2015, pp. 486). Thus, through a Baradian lens, we are prompted to see Western consumption practices and the continuing waste of activewear as another contributing factor to climate change and environmental injustices. While the cause-and-effect relationships may be hard-to-see in such complex entanglements, the feminist politics in Western women gaining strength and pride through their fitness (consumption) practices is negatively impacting the health and well-being of women in developing countries.

Recognizing the vitality of non-human matter is integral to environmental justice and necessary when looking for solutions that attend to the complexity of these most pressing issues. Barad describes that “what is entailed in matters of justice is paying careful attention to the ghosts in all their materiality—that is, all the labor, the really hard work, of tracing entanglements and being responsible to the liveliness of the world” (Barad in interview with Juelskjær and Schwennesen, 2012, pp. 22). Looking at the vitality of matter, at the “life” of activewear after it leaves the human helps to bring to light the impactful (and often unnoticed) role activewear clothing and women's consumption practices are having on the production of greenhouse gases.

## Conclusion

Baradian theory holds great potential for those interested in exploring the connection between sport and the environment. It was with agential realism and a diffractive research process that we came to think differently about women's embodied fitness and consumption practices. Reading different data sets and environmental and theoretical knowledge through each other, we were prompted to attend to the vitality of activewear clothing and its various environmental entanglements. Using a Baradian approach, we offered two examples of activewear-environment entanglements. The first revealed the ways the fear of sweaty smelling activewear and discourses around “fit femininity” are entangled with the increased accumulation of microplastics in the ocean that are harming aquatic ecosystems and human and more-than-human health. In the second example, we explored how the disposal and “retiring of activewear,” even when being donated to secondhand clothing stores and friends, is an active component contributing to the increased production of carbon dioxide emissions. Through these examples, we emphasized the vitality of activewear, revealing how the “fine details” of fitness practices are entangled with the environment, as well as broader social structures such as late capitalism, and discourses of “fit femininity,” neoliberalism, and conspicuous consumption.

Importantly, being open to the vitality of matter creates possibilities for more just and equitable ways of knowing and living. Barad (2007) describes how “learning how to intra-act responsibly as part of the world means understanding that ‘we' are not the only active beings—though this is never justification for deflecting our responsibility onto others” (pp. 391). Rather than focusing primarily on human desires and future generations of humans, care needs to be taken to appreciate non-human matter—oceans, animals, trees—and how some non-human matter (i.e., clothing, sporting, and fitness objects) is part of many (damaging) environmental entanglements. Within women's fitness practices, this means focusing on human experience *and* objects of fitness (i.e., clothing); what happens when we (knowingly and unknowingly) buy into cultures of conspicuous consumption (even when these purchases are aiding in the production of “healthier” neoliberal citizens)? What are the many human and non-human entanglements of activewear involved in the production process, before it “meets” its wearer? What are the more-than-human ethical considerations we should embark upon before consuming a new pair of leggings or sports bra? What are the broader effects of our compulsive desires to wash activewear after each usage (to meet feminine norms and avoid the fear of being the “stinky one”)? Where do our clothes go when we get “rid” of them? What form did this fabric take before it was made into a sports bra or a pair of leggings, and what is the process of its decomposition? While a number of sports scholars have embarked on political ecological and feminist environmental examinations of sport production, consumption, and participation practices, we argue that a Baradian approach is also valuable in that it encourages us to ask different sets of questions, to embark on theory-method experiments in tracing entanglements of non-human matter in physical cultural phenomena—stadiums, shoes, water, protein, grass, sand—and how these non-human objects are critically situated in larger ecologies and systems.

As Indigenous and feminist environmental scholars have long argued, and new materialist approaches are increasingly recognizing, acknowledging the entangled nature of the world has great potential for developing new policies that can provide different solutions for tackling some of the world's major issues, including environmental degradation. Currently, politics and policies targeting environmental degradation often emphasize the role of individuals or of humankind, more broadly, with greater emphasis on the production and manufacturing of consumer objects (i.e., clothing). While there are certainly human actions that can be done to minimize environmental impact and many changes required in the processes of manufacturing, such humanist approaches fail to recognize “a whole array of very complex material practices that contribute to a kind of epidemic [or catastrophe] that is not attributable either to the organisms [and non-human matter] themselves or to the kinds of things that people do” (Barad in interview with Juelskjær and Schwennesen, 2012, pp. 56). Instead, a mindset shift is needed—an ontological reorientation—toward the multiple ways that objects, humans, non-humans are connected. From our experiences of working with Barad's agential realism, we see much value and potential in ontological reorientations that encourage scholars to recognize these connections and complexities where the “fine details” of everyday practices come to *matter*.

In the context of women's fitness consumption practices, a Baradian onto-epistemology encourages new ways of understanding activewear as an active participant in the world's becoming (or unbecoming) and part of multiple entanglements that are agentic, that *do* something. Rethinking the activewear phenomenon as more-than-human phenomenon might encourage women to reconsider their consumption practices, as well as their laundering, disposal, and recycling of such luxury items. The small act of washing smelly activewear is entangled with broader discourses of “fit femininity,” neoliberalism and healthism, whilst also entangled with increase of dangerous microplastics in the ocean and various human and non-human bodies. As Indigenous and feminist environmental scholars have argued for many years, Western consumption practices are never isolated from the environment and climate change, with systems of gender, race and class oppression intimately tied to colonialism, capitalism, and issues of environmental justice. As this paper has shown, women's everyday fitness consumption practices are complicit in such processes.

Policies, politics, and solutions to environmental degradation need to be revised to better address the complexities of these more-than-human entanglements. This is no easy task and we concur with King and Weedon (2021) who describe how they have been “stretched and confounded in [their] efforts to recognize how complex histories of injustice and resilience are infused in biological matter, bodies, and multispecies traffic” (pp. 4). It is difficult, both ontologically and empirically, to think through new materialist implications and the very many entanglements of sport, the environment, and our own and others moving bodies. However, the struggle is worth it as we believe new materialisms (with other monistic ontologies), hold great potential for encouraging different ways of thinking about one of the most urgent global crises affecting all us (Braidotti, 2019). Through encouraging theory-method experiments toward knowing the entangled nature of *all* intra-actions—including our much loved sporting and fitness practices—agential realism offers alternative possibilities for doing research, and ways of knowing and doing differently, such that we can “use our ability to respond,” and it is thus “our responsibility, to help awaken, to breathe life into new possibilities for living justly.” (Barad, 2007, pp. x).

## Data Availability Statement

The datasets presented in this article are not readily available because the dataset includes confidential transcripts of interviews conducted with women in Aotearoa New Zealand. Requests to access the datasets should be directed to Julie E. Brice, jubrice@gmail.com.

## Ethics Statement

As this study involved human participants, it was reviewed and approved by the University of Waikato, Human Research Ethics Committee. The participants provided their written informed consent to participate in this study.

## Author Contributions

This paper was produced using research from JB's doctoral dissertation. JB was responsible for collecting and analyzing data, in addition to leading the writing of this paper. HT provided guidance and support during data collection and was instrumental in outlining and developing this paper. HT provided extensive editing during the writing phase. Both authors contributed to the article and approved the submitted version.

## Conflict of Interest

The authors declare that the research was conducted in the absence of any commercial or financial relationships that could be construed as a potential conflict of interest.

## Footnotes

^1^There are a variety of monikers for activewear: sportswear, athleisure, fitness wear. In this paper, we use activewear and athleisure interchangeably, although recognize that there has been debate about their synonymy.

^2^Healthism, as a concept, is most often attributed to Crawford (1980). In response to the various movements of self-care and holistic care in the 1970s, combined with neoliberalism in the 1980s, health became understood and situated at the level of the individual.

^3^All participant names are pseudonyms.

## References

[B1] Ahmed F. Pretorius L. (2010). Mega-events and environmental impacts: the 2010 FIFA World Cup in South Afirca. Alternation 17, 274–296.

[B2] Alaimo S. (2010). Material engagements: science studies and the environmental humanities. Ecozone 1, 69–74. 10.37536/ECOZONA.2010.1.1.322

[B3] Alaimo S. (2016). Environmental Politics and Pleasures in Posthuman Times. Minneapolis, MN: University of Minnesota Press.

[B4] Alaimo S. Hekman S. (2008). Introduction: emerging models of materiality in feminist theory, in Material *F*eminisms, eds Alaimo S. Hekman S. (Bloomington, IN: Indiana University Press), 1–19.

[B5] Anguelov N. (2015). The Dirty Side of the Garment Industry: Fast Fashion and its Negative Impact on Environment and Society. Boca Raton: CRC Press. 10.1201/b18902

[B6] Arora-Jonsson S. (2011). Virtue and vulnerability: discourses on women, gender and climate change. Global Environ. Change 21, 744–751. 10.1016/j.gloenvcha.2011.01.005

[B7] Atkinson M. (2014). The Terrier [Men]. Sociol. Sport J. 31, 420–437. 10.1123/ssj.2014-0089

[B8] Atkinson M. (2017). Ethnoaesthesia: ashtanga yoga and the sensuality of sweat, in Seeking the Senses in Physical Culture: Sensuous *S*cholarship in *A*ction, ed Sparkes A.C. (London: Routledge), 63–81. 10.4324/9781315657585-4

[B9] Azzarito L. (2019). Social Justice in Globalized Fitness and Health: Bodies Out of Sight. New York, NY: Roultedge. 10.4324/9781315163314

[B10] Baier D. Rausch T. Wagner T. (2020). The drivers of sustainable apparel and sportswear consumption: a segmented kano perspective. Sustainability 12, 2788–2809. 10.3390/su12072788

[B11] Bain M. (2020). Activewear is a bright spot in Covid-19 fashion sales. QUARTZ [Internet]. Available online at: https://www.msn.com/en-us/money/companies/activewear-is-a-bright-spot-in-covid-19-fashion-sales/ar-BB16PbdK (accessed December 15, 2020).

[B12] Barad K. (2003). Posthumanist performativity: toward an understanding of how matter comes to matter. Signs 28, 801–831. 10.1086/345321

[B13] Barad K. (2007). Meeting the Universe Halfway: Quantum Physics and the Entanglement of Matter and Meaning. Durham, NC: Duke University Press. 10.1215/9780822388128

[B14] Barad K. (2012). Intra-actions, in Mousse Magazine, ed Kleinman A., 76–81.

[B15] Bartley T. Child C. (2014). Shaming the corporation: the social production of targets and the anti-sweatshop movement. Am. Sociol. Rev. 79, 653–679. 10.1177/0003122414540653

[B16] Bastian M. Jones O. Moore N. Roe E. (eds.). (2017). Participatory Research in More-Than-Human Worlds. London: Routledge. 10.4324/9781315661698

[B17] Berners-Lee M. Clark D. (2010). What's the carbon footprint of.a load of laundry?. The Guardian [Internet]. Available online at: https://www.theguardian.com/environment/green-living-blog/2010/nov/25/carbon-footprint-load-laundry (accessed December 15, 2020).

[B18] Booth A. (2020). Entangling corporeal matter and geomatter: making and remaking the beach, in Sport, Physical Culture and the Moving Body: Materialisms, Technologies, and Ecologies, eds Newman J. Thorpe H. Andrews D. (New Brunswick, NJ: Routledge University Press), 246–67.

[B19] Boykoff J. Mascarenhas G. (2016). The Olympics, sustainability, and greenwashing: the Rio 2016 Summer Games. Capital. Nat. Social. 27, 1–11. 10.1080/10455752.2016.1179473

[B20] Braidotti R. (2019). Posthuman Knowledge. Cambridge: Polity Press.

[B21] Brian R. (2019). More than ever, our clothes are made of plastic. Just washing them can pollute the oceans. Vox [Internet]. Available online at: https://www.vox.com/the-goods/2018/9/19/17800654/clothes-plastic-pollution-polyester-washing-machine (accessed June 3, 2020).

[B22] Brice J. Thorpe H. (2021a). Activewear: the uniform of the neoliberal female citizen, in Sportswomen's *A*pparel *A*round the *W*orld: Uniformly *D*iscussed, ed Fuller L. K. (Cham: Springer International Publishing), 19–35. 10.1007/978-3-030-46843-9_2

[B23] Brice J. E. Clark M. Thorpe H. (2020). Feminist collaborative becomings: an entangled process of knowing through fitness objects. Qual. Res. Sport Exerc. Health. 10.1080/2159676X.2020.1820560

[B24] Brice J. E. Thorpe H. (2021b). The lively intra-actions of athleisure: A Baradian analysis of fit femininity. Somatechnics 11, 228–245.

[B25] Brymer E. Gray T. (2010). Developing an intimate “relationship” with nature through extreme sports participation. Leisure/Loisir 34, 361–374. 10.1080/14927713.2010.542888

[B26] Bunds K. Casper J. (2018). Sport, physical culture, and the environment: an introduction. Sociol. Sport J. 35, 1–7. 10.1123/ssj.2018-0007

[B27] Carrington D. (2017). Earth's sixth mass extinction event under way, scientists warn. The Guardian [Internet]. Available online at: https://www.theguardian.com/environment/2017/jul/10/earths-sixth-mass-extinction-event-already-underway-scientists-warn (accessed June 05, 2020).

[B28] Casper J. Pfahl M. E. (eds.). (2015). Sport Management and the Natural Environment: Theory and Practice. New York, NY: Routledge. 10.4324/9781315881836

[B29] Celermajer D. Chatterjee S. Cochrane A. Fishel S. Neimanis A. O'Brien A.. (2020). Justice through a multispecies lens. Crit. Exchange. 19, 475–512. 10.1057/s41296-020-00386-5

[B30] Chitrakorn K. (2017). Global sportswear brands making a play for women. The Business of Fashion [Internet]. Available online at: https://www.businessoffashion.com/articles/intelligence/how-sportswear-brands-are-making-a-play-for-women (accessed June 01, 2020).

[B31] Clary-Lemon J. (2019). Gifts, ancestors, and relations: notes toward an Indigenous new materialism. Enculturation.

[B32] Classen C. Howes D. Synnott A. (1994). Aroma: The Cultural History of Smell. Abingdon: Routledge.

[B33] Cobbing M. Brodde K. (2014). A Red Card for Sportswear Brands. Hamburg: Greenpeace.

[B34] Collins A. Jones C. Munday M. (2009). Assessing the environmental impacts of mega sporting events: two options? Tourism Manage. 30, 828–837. 10.1016/j.tourman.2008.12.006

[B35] Coole D. Frost S. (2010). Introducing the new materialisms, in New Materialisms: Ontology, Agency and Politics, eds Coole D. Frost S. (Durham, NC: Duke University Press), 1–43. 10.1215/9780822392996-001

[B171] Crawford R. (1980). Healthism and the medicalization of everyday life. Int. J. Health Serv. 10, 365–388. 10.2190/3H2H-3XJN-3KAY-G9NY 7419309

[B36] Cushman J. (1998, May 13). Nike pledges to end child labor and apply U.S. rules abroad. New York Times [Internet], D1.11648085

[B37] Dalton R. (2017). If you care about the environment and you should care about where your activewear comes. Well Made [Internet]. Available online at: https://wellmadeclothes.co.nz/articles/YouShouldCareAboutWhereYourActivewearComesFrom/ (accessed January 08, 2020).

[B38] Dashper K. (2019). Moving beyond anthropocentrism in leisure research: multispecies perspectives. Ann. Leis. Res. 22, 133–139. 10.1080/11745398.2018.1478738

[B39] De-la-Torre G. E. (2020). Microplastics: an emerging threat to food security and human health. J. Food Sci. Technol. 57, 1601–1608. 10.1007/s13197-019-04138-1 32327770 PMC7171031

[B40] Denton F. (2002). Climate change vulnerability, impacts, and adaptation: why does gender matter? Gend. Dev. 10, 10–20. 10.1080/13552070215903

[B41] Dirty Laundry (2011). Unravelling the Corporate Connections to Toxic Water Pollution in China. Amsterdam: Greenpeace International.

[B42] Dittmar H. (2011). Material and consumer identities, in Handbook of Identity Theory and Research, eds Schwartz S. J. Luyckx K. Vignoles V. L. (New York, NY: Springer New York), 745–769. 10.1007/978-1-4419-7988-9_31

[B43] Dohms E. (2019). Expert: throwing away clothing does serious damage to environment. Wisconsin Public Radio National Public Radio.

[B44] Dolphijn R. van der Tuin I. (2012). New Materialism: Interviews and Cartographies. Ann Arbor, MI: Open Humanities Press. 10.3998/ohp.11515701.0001.001

[B45] Driver R. (2020). Global activewear market set to be worth $353.5 billion in 2020. Fashion Network [Internet]. Available online at: https://uk.fashionnetwork.com/news/Global-activewear-market-set-to-be-worth-353-5-billion-in-2020,1266665.html (accessed January 05, 2021).

[B46] Dworkin S. Messner M. A. (2002). Just do.what? Sport, bodies, gender, in Gender and *S*port: A *R*eader, eds Scraton S. Flintoff A. (London: Routledge), 17–29.

[B47] Erdnüß L. (2016). A perspective on sustainability initiatives of a swedish outdoor brand—an interview with Lennart Ekberg from Haglöfs. Fashion Practice 8, 318–334. 10.1080/17569370.2016.1215115

[B48] Evers C. W. (2019a). Polluted Leisure. Leis. Sci. 41, 423–440. 10.1080/01490400.2019.1627963

[B49] Evers C. W. (2019b). Pollution, leisure, and blue space: more-than-human concerns in Fukushima. J. Sport Soc. Issues. 45, 179–195. 10.1177/0193723519884854

[B50] Falk M. (2020). How to shop for sustainable activewear. Shape [Internet]. Available online at: https://www.shape.com/fitness/clothes/how-to-shop-for-sustainable-activewear (accessed January 15, 2021).

[B51] Fox N. Alldred P. (2015). New materialist social inquiry: designs, methods and the research-assemblage. Int. J. Soc. Res. Methodol. 18, 399–414. 10.1080/13645579.2014.921458

[B52] Fox N. Alldred P. (2020a). Re-assembling climate change policy: materialism, posthumanism, and the policy assemblage. Br. J. Sociol. 71, 269–283. 10.1111/1468-4446.12734 31950493

[B53] Fox N. Alldred P. (2020b). Sustainability, feminist posthumanism and the unusual capacities of (post)humans. Environ. Sociol. 6, 121–131. 10.1080/23251042.2019.1704480

[B54] Fox N. Alldred P. (2018). New materialism, in The Sage Encyclopedia of Research Methods, eds Atkinon P. A. Delamont S. Hardy M. A. Williams M. (London: SAGE).

[B55] Ganak J. Summers L. Adesanya O. Chi T. Tai Y. (2019). The future of fashion sustainability: a qualitative study on U. S. female millennials' purchase intention towards sustainable synthetic athleisure apparel, in International Textile and Apparel Association Annual Conference Proceeding 76 (Las Vegas, NV). 10.31274/itaa.8237

[B56] Geeraert A. Gauthier R. (2018). Out-of-control Olympics: why the IOC is unable to ensure an environmentally sustainable Olympic Games. J. Environ. Policy Plann. 20, 16–30. 10.1080/1523908X.2017.1302322

[B57] Gough N. (2016). Postparadigmatic materialisms: a “new movement of thought” for outdoor environmental education research? J. Outdoor Environ. Educ. 19, 51–65. 10.1007/BF03400994

[B58] Greenberg J. Knight G. (2004). Framing sweatshops: nike, global production, and the American news media. Commun. Crit. Cult. Stud. 1, 151–175. 10.1080/14791420410001685368

[B59] Haaksluoto E. (2020). Exploring Instagram Influencers' Sportswear Endorsements and Consumer-Influencer Minteraction From Young Females' Perspective. Helsinki: Hanken University.

[B60] Hamilton C. (2010). Consumerism, self-creation and prospects for a new ecological consciousness. J. Cleaner Prod. 18, 571–575. 10.1016/j.jclepro.2009.09.013

[B61] Hauff C. R. (2016). Dress to impress or dress to sweat? examining the perceptions of exercise apparel through the eyes of active women. Women Sport Phys. Activ. J. 24, 99–109. 10.1123/wspaj.2015-0015

[B62] Hayes G. Karamichas J. (eds.). (2012). Olympic *G*ames, *M*ega-*E*vents and *C*ivil *S*ocieties: Globalization, *E*nvironment, *R*esistance. New York, NY Palgrave Macmillan. 10.1057/9780230359185

[B63] Hein S. (2016). The new materialism in qualitative inquiry: how compatible are the philosophies of Barad and Deleuze? Cult. Stud. Crit. Methodol. 16, 132–140. 10.1177/1532708616634732

[B64] Henhawk D. Norman R. (2019). ‘Indigenous peoples, sport and sustainability,” in Sport, *D*evelopment, and *E*nvironmental *S*ustabinility, eds Millington R. Darnell S. (New York, NY: Routledge), 163–177. 10.4324/9781351128629-11

[B65] Herweyers L. Carteny C. Scheelen L. Watts R. Els D. B. (2020). Consumers' perceptions and attitudes toward products preventing microfiber pollution in aquatic environments as a result of the domesticwashing of synthetic clothes. Sustainability 12, 1–14. 10.3390/su12062244

[B66] Hill L. L. Abbott A. (2009). Surfacing tension: toward a political ecological critique of surfing representations. Geography Compass 3, 275–296. 10.1111/j.1749-8198.2008.00192.x

[B67] Hird M. J. (2012). Knowing waste: towards an inhuman epistemology. Soc. Epistemol. 26, 453–469. 10.1080/02691728.2012.727195

[B68] Højgaard L. Søndergaard D. M. (2011). Theorizing the complexities of discursive and material subjectivity: agential realism and poststructural analyses. Theory Psychol. 21, 338–354. 10.1177/0959354309359965

[B69] Horan R. (2011). Where's the Buzz? Why No *One is Talking About lululemon athletica's Sustainability Initiatives*. Manitoba: University of Manitoba.

[B70] Horton K. Ferrero-Regis T. Payne A. (2016). The hard work of leisure: healthy life, activewear and Lorna Jane. Ann. Leis. Res. 19, 180–193. 10.1080/11745398.2015.1111149

[B71] Humberstone B. (2011). Embodiment and social action in nature-based sport: spiritual spaces. Leis. Stud. 30, 495–512. 10.1080/02614367.2011.602421

[B72] Johnson C. E. (2016). Nike Becomes a Global Citizen: London:SAGE.

[B73] Josephson P. R. (2015). Fish Sticks, Sports Bras, and Aluminum Cans: The Politics of Everyday Technologies. Baltimore, MD: Johns Hopkins University Press.

[B74] Juelskjær M. Schwennesen N. (2012). Intra-active entanglements: an interview with Karen Barad. Kvinder Køn og Forskning 1, 10–24. 10.7146/kkf.v0i1-2.28068

[B75] Karamichas J. (2013). The Olympic Games and the Environment. New York, NY: Palgrave Macmillan. 10.1057/9781137297471

[B76] Karanjekar R. V. Bhatt A. Altouqui S. Jangikhatoonabad N. Durai V. Sattler M. L.. (2015). Estimating methane emissions from landfills based on rainfall, ambient temperature, and waste composition: The CLEEN model. Waste Manage. 46, 389–398. 10.1016/j.wasman.2015.07.030 26346020

[B77] Kay J. (2017, March 15). The trendy athleisure clothing everyone is wearing is harming the environment. Business Insider [Internet]. Available online at: https://www.businessinsider.com/athleisure-clothing-could-be-damaging-the-environment-2017-3?IR=T (accessed December 15, 2020).

[B78] King S. (2020). Towards a multispecies sport studies, in Sport, Physical Culture and the Moving Body: Materialisms, Technologies and Ecologies, eds Newman J. Thorpe H. Andrews D. (New Brunswick, NJ: Rutgers University Press), 193–208.

[B79] King S. Weedon G. (2020). Embodiment is ecological: the metabolic lives of whey protein powder. Body Society 26, 82–106. 10.1177/1357034X19878775

[B80] King S. Weedon G. (2021). The nature of the body in sport and physical culture: bodies and environments to ecological embodiment. Sociol. Sport J. 10.1123/ssj.2020-0038

[B81] Kiuchi Y. (2021). Fetishization of women's athletic wear: Japanese obsessions with bloomers, in Sportswomen's *A*pparel *A*round the *W*orld; *U*niformly *D*iscussed. New *F*emininites in *D*igital, *P*hysical and *S*porting *C*ultures, ed Fuller L. (London: Palgrave Macmillan), 131–150. 10.1007/978-3-030-46843-9_9

[B82] Knight G. Greenberg J. (2002). Promotionalism and subpolitics: nike and its labor critics. Manage. Commun. Q. 15, 541–570. 10.1177/0893318902154002

[B83] Koro-Ljungberg M. (2015). Reconceptualizing Qualitative Research: Methodoloiges Without Methodology. Los Angeles, CA: SAGE Publications. 10.4135/9781071802793

[B84] Kosuth M. Mason S. Wattenberg E. (2018). Anthropogenic contamination of tap water, beer, and sea salt. PLoS ONE 13:0194970. 10.1371/journal.pone.0194970 29641556 PMC5895013

[B85] Laurendeau J. (2020). ‘The stories that will make a difference aren't the easy ones': outdoor recreation, the wilderness ideal, and complicating settler mobility. Sociol. Sport J. 37, 85–95. 10.1123/ssj.2019-0128

[B86] Lavrence C. Lozanski K. (2014). 'This is not your practice life': Lululemon and the neoliberal governance of self. Can. Rev. Sociol. 51, 76–94. 10.1111/cars.12034

[B87] Lemke T. (2014). New materialisms: foucault and the ‘government of things'. Theory Cult. Soc. 32, 3–25. 10.1177/0263276413519340

[B88] Lenskyj H. J. (1998). Sport and corporate environmentalism: the case of the sydney 2000 olympics. Int. Rev. Sociol. Sport 33, 341–354. 10.1177/101269098033004002

[B89] Linghede E. (2018). The promise of glitching bodies in sport: a posthumanist exploration of an intersex phenomenon. Qual. Res. Sport Exerc. Health 10, 570–584. 10.1080/2159676X.2018.1479980

[B90] Lipson S. M. Stewart S. Griffiths S. (2020). Athleisure: a qualitative investigation of a multi-billion-dollar clothing trend. Body Image 32, 5–13. 10.1016/j.bodyim.2019.10.009 31756602

[B91] LuLuLemon (2018). Available online at: https://www.lululemon.co.nz/ (accessed January 18, 2021).

[B92] MacLure M. (2013a). The wonder of data. Cult. Stud. Crit. Methodol. 13, 228–232. 10.1177/1532708613487863

[B93] MacLure M. (2013b). Classification of wonder? Coding as an analytic practice in qualitative research, in Deleuze and Research Methodologies, eds Coleman B. Ringrose J. (Edinburgh: Edinburgh University Press), 164–183.

[B94] Malacoff J. (2017). Here's the real reason why you shouldn't wear dirty workout clothes. Brtico [Internet]. Available online at: https://www.brit.co/heres-why-you-shouldnt-wear-dirty-workout-clothes/ (accessed December 18, 2020).

[B95] Mansfield L. (2009). Fitness cultures and environmental (in)justice? Int. Rev. for the Sociol. Sport 44, 345–362. 10.1177/1012690209343029

[B96] Markula P. (2019). What is new about new materialism for sport sociology? Reflections on body, movement and culture. Sociol. Sport J. 36:64. 10.1123/ssj.2018-0064

[B97] Masanell-Casadesus R. Crooke M. Reinhardt F. Vasisth V. (2009). Households' willingness to pay for “green” goods: evidence from patagonia's introduction of organic cotton sportswear. J. Econ. Manage. Strategy 18, 203–233. 10.1111/j.1530-9134.2009.00212.x

[B98] Matera A. (2019). Because finding sustainable activewear shouldn't be harder than your workout—here are 8 places to start. Available online at: https://www.wellandgood.com/good-looks/sustainable-activewear-brands/ (accessed January 18, 2021).

[B99] McCarthy J. (2020). Why climate change disproportionately affects women Global Citizen. Available online at: https://www.globalcitizen.org/en/content/how-climate-change-affects-women/ (accessed December 08, 2020).

[B100] McCoy J. (2019). How gross is it really to re-wear sweaty workout clothes? Self [Internet]. Available online at: https://www.self.com/story/how-gross-re-wear-sweaty-workout-clothes (accessed January 10, 2020).

[B101] McCullough B. Kellison T. (eds.). (2017). Routledge Handbook of Sport and the Environment. London: Routledge. 10.4324/9781315619514

[B102] McDonald M. G. Sterling J. (2020). Feminist new materialisms and the trouble watrs of the 2016 Rio de Janeiro Olympic and Paralympic Games, in Sport, Physical Culture and the Moving Body: Materialisms, Technologies and Ecologies, eds Newman J. Thorpe H. Andrews D. (New Brunswick, NJ: Rutgers University Press), 283–300.

[B103] McKnight L. (2016). Swimming lessons: learning, new materialisms, posthumanism, and post qualitative research emerge through a pool poem. J. Curricul. Pedagogy 13, 195–205. 10.1080/15505170.2016.1220875

[B104] Merchant S. (2020). Running with an ‘other': landscape negotitation and inter-relationality in canicross. Sport Soc. 23, 11–23. 10.1080/17430437.2018.1555212

[B105] Mezzadri A. (2016). Class, gender and the sweatshop: on the nexus between labour commodification and exploitation. Third World Q. 37, 1877–1900. 10.1080/01436597.2016.1180239

[B106] Millington B. Wilson B. (2017). Contested terrain and terrain that contests: Donald Trump, golf's environmental politics, and a challenge to anthropocentrism in Physical Cultural Studies. Int. Rev. Sociol. Sport 52, 910–923. 10.1177/1012690216631541

[B107] Mishra S. Rath C.c Das A. P. (2019). Marine microfiber pollution: a review on present status and future challenges. Mar. Pollut. Bull. 140, 188–197. 10.1016/j.marpolbul.2019.01.039 30803634

[B108] Moon V. (2017). Can sportswear make sustainability cool? Huffpost [Internet]. Available online at: https://www.huffpost.com/entry/is-sportswear-the-key-to_b_12274188 (accessed January 01, 2021).

[B109] Nam C. Dong H. Lee Y.-A. (2017). Factors influencing consumers' purchase intention of green sportswear. Fashion Textiles 4:2. 10.1186/s40691-017-0091-3

[B110] Nash M. (2016). Selling health and fitness to sporty sisters: a critical feminist multi-modal discourse analysis of the Lorna Jane retail website. Sociol. Sport J. 33, 219–229. 10.1123/ssj.2015-0105

[B111] Neimanis A. (2016). Bodies of Water: Posthuman Feminist Phenomenology London: Bloomsbury Academic.

[B112] Neimanis A. Asberg C. Hayes S. (2015). Posthumanist imaginaries, in Research *H*andbook on *C*limate *G*overnance, eds Backstrom K. Lovbrand E. (Northhamption: Edward Elgar), 480–490. 10.4337/9781783470600.00055

[B113] Nike (2021). Human Rights and Labor Compliance Standards: Nike. Available online at: https://purpose.nike.com/human-rights (accessed January 15, 2021).

[B114] Nordstrom S. (2018). Antimethodology: postqualitative generative conventions. Qual. Inquiry 24, 215–226. 10.1177/1077800417704469

[B115] Norgaard K. M. (2012). Climate denital and the construction of innocence: reproducing transnational environmental privilege in the face of climate change. Race Gender Class 19, 80–103.

[B116] Norman M. E. Hart M. Mason G. (2020). Restor(y)ing place: indigenous land-based physical cultural practices as restorative process in fisher river cree nation (Ochékwi Sipi), in Sport and the Environment. Research in the Sociology of Sport, eds Wilson B. Millington B. (Bingley: Emerald Publishing Limited), 85–101. 10.1108/S1476-285420200000013005

[B117] Ocean Clean Wash (2019). Microfiber pollution through washing and wearing. Ocean Clean Wash. Available online at: https://www.oceancleanwash.org/ (accessed December 14, 2020).

[B118] Olive R. (2016). Surfing, localism, place-based pedagogies, and ecological sensibilities in Australia, in Routledge International Handbook of Outdoor Studies, eds Humberstone B. Prince H. Henderson K. A. (Abingdon: Routledge), 501–510. 10.4324/9781315768465-56

[B119] Olive R. (2019). The trouble with newcomers: women, localism and the politics of surfing. J. Aust. Stud. 43, 39–54. 10.1080/14443058.2019.1574861

[B120] Olive R. Wheaton B. (2021). Understanding blue spaces: sports, bodies, wellbeing, and the sea. J. Sport Soc. Issues. 45, 3–19. 10.1177/0193723520950549

[B121] O'Loughlin C. (2018). The impact of activewear/swimwear laundering: Investigating microplastic- fibre emissions from recycled and non-recycled synthetic textiles. J. HEIA 25, 2–12.

[B122] ÖzdIl N. Anand S. (2014). Recent development in textile materials and products used for activewear and sportswear. Elec. J. Textile Technol. 8, 68–83.

[B123] Patagonia.com (2020). Available online at: https://www.patagonia.co.nz/ (accessed January 18, 2021).

[B124] Petrina S. (2001). A cultural study and political ecology of nike “air jordans”. Int. J. Technol. Design Educ. 10, 207–237. 10.1023/A:1008955016067

[B125] Petter O. (2020). Sustainable activewear: how your gym kit is going green. Independent [Internet]. Available online at: https://www.independent.co.uk/life-style/fashion/sustainable-activewear-gym-green-eco-friendly-carbon-footprint-a9287131.html (accessed January 15, 2021).

[B126] Pierides D. Woodman D. (2012). Object-oriented sociology and organizing in the face of emergency: Bruno Latour, Graham Harman and the material turn. Br. J. Sociol. 63, 662–679. 10.1111/j.1468-4446.2012.01431.x 23240837

[B127] Pinch T. (2011). Karen Barad, quantum mechanics, and the paradox of mutual exclusivity. Soc. Stud. Sci. 41, 431–441. 10.1177/0306312711400657

[B128] Prata J. C. da Costa J. P. Lopes I. Duarte A. C. Rocha-Santos T. (2020). Environmental exposure to microplastics: an overview on possible human health effects. Sci. Total Environ. 702:134455. 10.1016/j.scitotenv.2019.134455 31733547

[B129] Ráthonyi G. Ráthonyi-Odor K. (2015). Analysing sporting goods manufacturers' environmental management tools. Appl. Stud. Agribusiness Commerce 9, 23–30. 10.19041/APSTRACT/2015/1-2/5

[B130] Ravenscroft A. (2018). Strange weather: indigenous materialisms, new materialism, and colonialism. Cambridge J. Postcolonial Literary Inquiry 5, 353–370. 10.1017/pli.2018.9

[B131] Rosiek J. L. Snyder J. Pratt S. L. (2019). The new materialisms and indigenous theories of non-human agency: making the case for respectful anti-colonial engagement. Qual. Inquiry 26, 1–16. 10.1177/1077800419830135

[B132] Ross P. (2019). Every time you wash clothes, millions of microfibers are released into the water, in National Public Radio, ed Hobson J. (Boston, MA).

[B133] Saied N. A. Creedon P. (2021). Women's sports and fashion in Arab Gulf countries, in Sportswomen's *A*pparel *A*round the *W*orld; *U*niformly *D*iscussed. New *F*emininites in *D*igital, *P*hysical and *S*porting *C*ultures, ed Fuller L. (London: Palgrave Macmillan), 69–82. 10.1007/978-3-030-46843-9_5

[B134] Schmidt J. (2013). The empirical falsity of the human subject: new materialism, climate change and the shared critique of artifice. Resilience 1, 174–192. 10.1080/21693293.2013.837241

[B135] Shove E. (2003). Comfort, Cleanliness and Convenience: The Social Organization of Normality. Oxford, UK: Berg.

[B136] Stoddart M. (2012). Making Meaning Out of Mountains: The Political Ecology of Skiing. Vancouver: BC Press.

[B137] St. Pierre E. (2011). Post qualitative research: the critique and the coming after, in The SAGE Handbook of Qualitative Research, eds Denzin N. Lincoln Y. (Los Angeles, CA: SAGE), 611–626.

[B138] Subic A. (2010). Sustainability and the sports industry. Sports Technol. 3:221. 10.1080/19346182.2010.693249

[B139] Subic A. Mouritz A. Troynikov O. (2009). Sustainable design and environmental impact of materials in sports products. Sports Technol. 2, 67–79. 10.1080/19346182.2009.9648504

[B140] Subic A. Paterson N. (2006). Life Cycle Assessment and Evaluation of Environmental Impact of Sports Equipment. The Engineering of Sport. New York, NY: Springer New York. 10.1007/978-0-387-45951-6_8

[B141] Subic A. Shabani B. Hedayati M. Crossin E. (2012). Capability framework for sustainable manufacturing of sports apparel and footwear. Sustainability 4, 2127–2145. 10.3390/su4092127

[B142] Sweaty Betty London Modern Slavery Statement (2019). Available online at: https://www.sweatybetty.com/on/demandware.static/-/Library-Sites-sweatybettylibrary/default/dw3b51d166/images/content/modernslavery/Modern_Slavery_Act_Statement_2019.pdf (accessed January 15, 2021).

[B143] Textiles Timaru N. Z. (2015). One planet-New Zealand. Available online at: https://www.oneplanet.org.nz/recycle/household-recycling/textiles (accessed December 15, 2020).

[B144] The Story of Stuff Project (2020). Available online at: https://www.storyofstuff.org/ (accessed December 10, 2020).

[B145] Thomas A. C. (2015). Indigenous more-than-humanisms: relationship ethics with the Hurunui River in Aotearoa New Zealand. Soc. Cult. Geogr. 16, 947–990. 10.1080/14649365.2015.1042399

[B146] Thorpe H. Brice J. E. Clark M. (2020). Feminist New Materialisms, Sport, and Fitness: A Lively Entanglement. Palgrave: Macmillan. 10.1007/978-3-030-56581-7

[B147] Thorpe H. Brice J. E. Clark M. (2021). New materialisms, sport and the environment: towards a research agenda. Sport Educ. Soc. 26, 363–377. 10.1080/13573322.2020.1837097

[B148] Todd Z. (2016). An indigenous feminist's take on the ontological turn: ‘ontology' is just another word for colonialism. J. Hist. Sociol. 29, 4–22. 10.1111/johs.12124

[B149] Trendafilova S. Babiak K. Heinze K. (2013). Corporate social responsibility and environmental sustainability: why professional sport is greening the playing field. Sport Manage. Rev. 16, 298–313. 10.1016/j.smr.2012.12.006

[B150] United States Environmental Protection Agency (2017). Facts and Figures About Materials, Waste and Recycling. Washington, DC: United States Environmental Protection Agency. Available online at: https://www.epa.gov/facts-and-figures-about-materials-waste-and-recycling/nondurable-goods-product-specific-data#ClothingandFootwear (accessed November 10, 2020).

[B151] van Luijk N. Giles A. R. Hayhurst L. M. C. (2020). Extractives industry and sport for development: how is right to play promoting environmental sustainability in Indigenous communities in Canada?, in Sport and the Environment, eds Wilson B. Millington R. (Bingley: Emerald Publishing Limited), 47–66. 10.1108/S1476-285420200000013003

[B152] Waiti J. Awatere S. (2019). Kaihekengaru: Māori surfers' and a sense of place. J. Coast. Res. 87, 35–43. 10.2112/SI87-004.1

[B153] Waitt G. Stanes E. (2015). Sweating bodies: men, masculinities, affect, emotion. Geoforum 59, 30–38. 10.1016/j.geoforum.2014.12.001

[B154] Wang L. Shen B. (2017). A product line analysis for eco-designed fashion products: evidence from an outdoor sportswear brand. Sustainability 9, 1–12. 10.3390/su9071136

[B155] Watts V. (2013). Indigenous place-thought and agency amongst humans and non-humans (First Woman and Sky Woman go on a European world tour!). Decolonization 2, 20–34.

[B156] Weekendbee (2019, November 30). Sustainability in the sportswear industry. Available online at: https://www.weekendbee.com/blogs/sustainability/what-is-sustainability-in-the-sportswear-industry (accessed December 15, 2020).

[B157] Wheaton B. (2020). Surfing and environmental sustainability, in Sport and the Environment (Research in the Sociology of Sport), Vol. 13, eds Wilson B. Millington B. (Emerald Publishing Limited), 157–178.

[B158] Wheaton B. Waiti J. Cosgriff M. Burrows L. (2019). Coastal blue space and wellbeing research: looking beyond western tides. Leis. Stud. 39, 83–95. 10.1080/02614367.2019.1640774

[B159] Wheeler K. Nauright J. (2006). A global perspective on the environmental impact of golf. Sport Soc. 9, 427–443. 10.1080/17430430600673449

[B160] Williams M. S. (2016). Strategic innovation in US anti-sweatshop movement. Soc. Mov. Stud. 15, 277–289. 10.1080/14742837.2015.1082466

[B161] Wilson B. Millington B. (2017). Physical Cultural Studies, Sport and the Environment. 1 Edn. London: Routledge, 333–343. 10.4324/9781315745664-35

[B162] Wilson B. Millington B. (eds.). (2020). Sport and the Environment. Bingley: Emerald Publishing Limited.

[B163] Woolf A.-L. (2019). New Zealand landfills are becoming full of unloved clothes as 'fast fashion' grows. Stuff.co.nz. Available online at: https://www.stuff.co.nz/environment/114298459/new-zealand-landfills-are-becoming-full-of-unloved-clothes-as-fast-fashion-grows (accessed December 11, 2020).

[B164] World Wildlife Fund Causes of Climate Change. Wellington NZ: World Wildlife Fund. Available online at: https://www.wwf.org.nz/what_we_do/climateaction/causes_of_climate_change/ (accessed December 01 2020).

[B165] Wright S. Kelly F. (2017). Plastic and human health: a micro issue? Environ. Sci. Technol. 51, 6634–6647. 10.1021/acs.est.7b00423 28531345

[B166] Wu J. X. Li l. (2019). Sustainability initiatives in the fashion industry. InTechOpen.

[B167] Yan S. Henninger C. Jones C. McCormick H. (2020). Sustainable knowledge from consumer perspective addressing microfibre pollution. J. Fashion Market. Manage. 24, 437–454. 10.1108/JFMM-08-2019-0181

[B168] Yates J. S. Harris L. M. Wilson N. J. (2017). Multiple ontologies of water: politics, conflict and implications for governance. Environ. Plann. D 35, 797–815. 10.1177/0263775817700395

[B169] Young I. (1990). Throwing Like a Girl and Other Essays in Feminist Philosophy and Social Theory Indianapolis, IN: Indiana University Press.

[B170] Zheng Y. Chi T. (2015). Factors influencing purchase intention towards environmentally friendly apparel: an empirical study of US consumers. Int. J. Fashion Design Technol. Educ. 8, 68–77. 10.1080/17543266.2014.990059

